# Prebiotic Potential of a New Sweetener Based on Galactooligosaccharides and Modified Mogrosides

**DOI:** 10.1021/acs.jafc.2c01363

**Published:** 2022-07-13

**Authors:** Ana Muñoz-Labrador, Rosa Lebrón-Aguilar, Jesús E. Quintanilla-López, Plácido Galindo-Iranzo, Silvana M. Azcarate, Sofia Kolida, Vasiliki Kachrimanidou, Virginia Garcia-Cañas, Lisa Methven, Robert A. Rastall, F. Javier Moreno, Oswaldo Hernandez-Hernandez

**Affiliations:** † Institute of Food Science Research, CIAL (CSIC-UAM), Nicolas Cabrera, 9, 28049 Madrid, Spain; ‡ Institute of Physical Chemistry “Rocasolano” (IQFR-CSIC), Serrano 119, 28006 Madrid, Spain; § Institute of Earth and Environmental Sciences of La Pampa (INCITAP), Mendoza 109, L6302EPA Santa Rosa, La Pampa, Argentina; ∥ OptiBiotix Health Plc, Innovation Centre, Innovation Way, Heslington, York YO10 5DG, U.K.; ⊥ Department of Food and Nutritional Sciences, The University of Reading, PO Box 226, Whiteknights, Reading RG6 6 AP, U.K.

**Keywords:** Siraitia grosvenorii, GOS, functional food, probiotic, sugar substitute

## Abstract

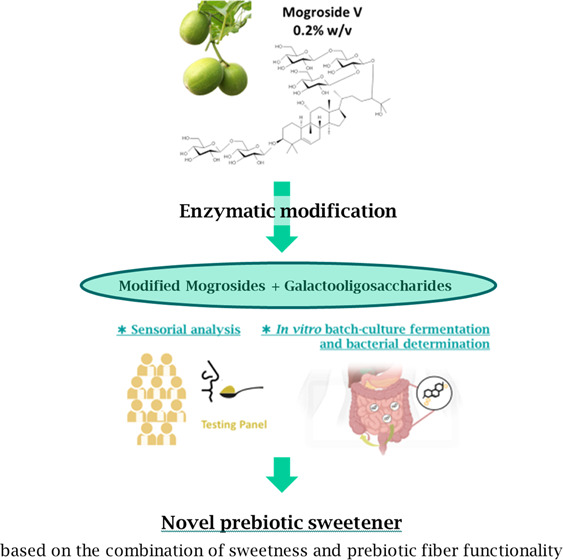

This study was conducted to investigate the sweetness intensity and the potential fecal microbiome modulation of galactooligosaccharides in combination with enzymatically modified mogrosides (mMV-GOS), both generated through a patented single-pot synthesis. Sweetness intensity was performed *in vivo* by trained sensory panelists. The impact on the human fecal microbiome was evaluated by *in vitro* pH-controlled batch fermentation, and bacterial populations and organic acid concentrations were measured by qPCR and GC-FID, respectively. Significant growth (*p* ≤ 0.05) during the fermentation at 10 h of bacterial populations includes *Bifidobacterium* (8.49 ± 0.44 CFU/mL), *Bacteroides* (9.73 ± 0.32 CFU/mL), *Enterococcus* (8.17 ± 0.42 CFU/mL), and *Clostridium coccoides* (6.15 ± 0.11 CFU/mL) as compared to the negative control counts for each bacterial group (7.94 ± 0.27, 7.84 ± 1.11, 7.52 ± 0.37, and 5.81 ± 0.08 CFU/mL, respectively) at the same time of fermentation. Likewise, the corresponding significant increase in production of SCFA in mMV-GOS at 10 h of fermentation, mainly seen in acetate (20.32 ± 2.56 mM) and propionate (9.49 ± 1.44 mM) production compared to a negative control at the same time (8.15 ± 1.97 and 1.86 ± 0.24 mM), is in line with a positive control (short-chain fructooligosaccharides; 46.74 ± 12.13 and 6.51 ± 1.91 mM, respectively) revealing a selective fermentation. In conclusion, these substrates could be considered as novel candidate prebiotic sweeteners, foreseeing a feasible and innovative approach targeting the sucrose content reduction in food. This new ingredient could provide health benefits when evaluated in human studies by combining sweetness and prebiotic fiber functionality.

## Introduction

The Food and Agriculture Organization of the United Nations (FAO) and the World Health Organization (WHO) describe carbohydrates as a major source of energy provided in the human diet, accounting for between 40 and 80% of the total energy requirements.^1^ Since the 1970s, specific health claims and recommendations have been steadily made around the world regarding the daily intake of dietary fibers due to the health benefits they provide.^2^ On the other hand, there is a growing concern about the global rise in diet-related health issues caused by excessive consumption of nutrients (mainly free sugars and fats), leading to imbalanced energy homeostasis and, consequently, the development of cardiovascular diseases, diabetes, gastrointestinal infections, obesity, and some forms of cancers, among others.^3−5^ The potential link between these diseases and the high intake of free sugars has been known for many years. Concerned over the potential adverse consequences, in 2015, several public health policies from the WHO, the Scientific Advisory Committee on Nutrition (SACN), and the Dietary Guidelines Advisory Committee (DGAC) recommended reducing the consumption of sugar to less than 5–10% of the total energy intake.^6−8^

Among the health benefits of dietary fibers, laxation, improvement of blood lipids, blood glucose regulation or mineral absorption, and the modulation of the immune system and satiety have long been appreciated.^9^ However, within the dietary fiber classification, certain nondigestible carbohydrates have attracted particular interest from the food industry as they play a positive effect on health such as prebiotics. Currently, prebiotic is defined as “a substrate that is selectively utilized by host microorganisms conferring a health benefit.”^10^ Galactooligosaccharides (GOS) are among the most commonly used prebiotics known to promote the growth of beneficial microorganisms, mainly intestinal lactobacilli and bifidobacteria, which can induce microbial competition and reduce the population of nonbeneficial intestinal microbiota.^11,12^ GOS are commercially available prebiotics, with a low calorific value, and are reported to be capable of promoting satiety and reducing food intake,^13^ as well as having clinical applications, including treatment of constipation and irritable bowel syndrome (IBS), prevention of atopic disease and gastrointestinal infections, or modulating mood and the stress response, among others.^14^ However, their sweetness properties are not suitable to be fully used as sucrose substitutes since one of the predominant factors contributing to sweetness is the degree of polymerization (DP) of carbohydrates which is inversely proportional. This means that the sweetness decreases with the increasing length of the oligosaccharide chain.^15,16^

As a result of the aforementioned diet-related health problems, there is a growing interest in the use of natural low and noncaloric sweeteners in food to reduce the free sugar intake. Among all of the recognized sweeteners, a natural-based extract from luo han guo fruit (*Siraitia grosvenorii*), formed by cucurbitane-type triterpenoid saponins known as mogrosides (mainly mogroside V), has gained the attention of consumers and the industrial sector. Mogroside V could be a potential candidate for replacing sugars; it is 200–300 times sweeter than sucrose. However, one of the main organoleptic issues with these compounds is the presence of off-flavors, such as bitterness and metallic side taste.^17−19^ It has been noted that the enzymatic glycosylation of these terpenoids could improve their taste profile.^20,21^ Even when glycosylation improves the taste, the relative sweetness value is still high. Therefore, these products are still considered as high-intensity sweeteners (HIS) with the concomitant lack of bulk properties. Taking this into account, an approach that would utilize dietary fibers, more specifically, prebiotics in combination with natural HIS, could overcome these challenges since prebiotics are considered as bulking agents in food industry, while enriching the nutritional value and health functionality associated with these ingredients.^10,22^ On the other hand, it is important to bear in mind sugar guidelines such as the American Heart Association (AHA) guideline, which stated a limit of added sugar intake of 25 g per day for women and 36 g per day for men.^23^ Assuming that prebiotic-based component doses of 2–15 g per day were described to exert a prebiotic benefit to health,^24^ the use of a prebiotic ingredient as a sugar substitute would perfectly fit into the requirements for either exerting a prebiotic effect or complying with the recommended minimization of sugar calories.

Therefore, this work will look at the hypothesis that the synthesis of a new sweetener based on galactooligosaccharides and enzymatically modified mogrosides could provide enough prebiotic and sweetness properties to be considered as a promising low-calorie and functional ingredient with a high consumers’ acceptance. The results included in this work have been derived from a patented technology based on the one-pot enzymatic synthesis of modified mogroside V and GOS recently described,^25^ highlighting their novelty and scientific relevance. The synthesis is mediated by fungal β-galactosidases in the presence of lactose and mogroside V, which results in a mixture of GOS, deglycosylated and galactosylated mogroside V, and glucose and galactose, which are subsequently eliminated to decrease the caloric value of the final product.

The main objective of this work was to evaluate the effect of the enzymatically modified mogroside extract and GOS, obtained via a one-pot technology, on the human fecal microbiota and the sweetness value by determining the sucrose equivalent percentage.

## Materials and Methods

### Chemicals

The high-purity mogroside V (≥98%) standard was purchased from Biosynth Carbosynth (Reading, U.K.). Acetonitrile (MS grade) and formic acid (MS grade) were obtained from Sigma-Aldrich (St. Louis). Positive control samples such as short-chain fructooligosaccharides (scFOS; 95% purity) from FUJIFILM Wako Chemicals (Germany) and synthesized GOS (Optibiotix Health Plc, York, U.K.) were used. White granulated sugar (Tate and Lyle, London, U.K.) and water (Harrogate Spa mineral water) for sensory analysis were purchased from local supermarkets in Reading (U.K.). Commercial organic acids were purchased from Sigma-Aldrich (U.K.). All of the other reagents were obtained from Sigma-Aldrich (St. Louis) and Thermo Fisher Scientific (San Jose) and were analytical grade.

### Test Samples

Modified mogroside V (mMV-GOS) was obtained from Optibiotix Health plc (York, U.K.). According to the manufacturer, mMV-GOS has been obtained by the enzymatic synthesis of GOS by β-galactosidases in the presence of lactose and mogroside V.^25^ The product contains 0.2% (w:w) of total mogrosides and GOS ≥ 95%. The sample does not contain mono- or disaccharides as determined by gas chromatography with flame-ionization detection (GC-FID).^26^

In this study, the nonmodified mogroside extract mainly obtained from mogroside V (MV) was also included in the fermentation study.

### Structural Characterization by MALDI-TOF MS Analysis

Matrix-assisted laser desorption ionization time-of-flight spectra were obtained using a Voyager-DE PRO mass spectrometer (Applied Biosystems, Foster City) operating in linear mode. Positive ions were extracted with an accelerating voltage of 25 kV and a delay time of 400 ns. Grid and guidewire voltages were set to 94 and 0.075%, respectively. Mass spectra were recorded in the range *of m*/*z* 500–4000, detecting glycosylated species as [M + Na]^+^.

Samples were diluted 100-fold with water and mixed with the matrix solution (2,5-dihydroxybenzoic acid at 10 mg/mL in water) in an approximate ratio of 1:3 (v:v). One microliter of this solution was spotted onto a flat stainless-steel sample plate and dried in air before analysis. Mass spectra were calibrated externally using the average [M + H]^+^ values of the constituents of the calibration mixtures 1 and 2 (Sequazyme Peptide Mass Standards Kits, Applied Biosystems).

### *In Vitro* Batch-Culture Fermentations

*In vitro* fermentations were carried out using human fecal microbiota collected from four healthy donors (two males and two females, aged 26–36 years) with no preceding history of metabolic or gastrointestinal disorders and who had not taken prebiotics or probiotics for 1 month nor antibiotics within 6 months before the study. Fecal slurries were prepared at 10% (w:v) with sterilized phosphate-buffered saline (PBS; 0.1 M, pH 7.4, Oxoid, Basingstoke, U.K.) and homogenized in a stomacher (Stomacher 400, Seward, U.K.) at normal speed for 2 min.

Microscale, sterile-stirred batch-culture fermentation systems (20 mL of working volume) were aseptically filled with 17 mL of sterile, nutrient basal medium containing 2 g/L peptone water, 2 g/L yeast extract, 0.1 g/L NaCl, 0.04 g/L K_2_HPO_4_, 0.04 g/L KH_2_PO_4_, 0.01 g/L MgSO_4_·7H_2_O, 0.01 g/L CaCl_2_·6H_2_O, 1 g/L NaHCO_3_, 0.5 g/L l-cysteine hydrochloride, 0.5 g/L bile salts, 0.05 g/L hemin, 10 μL/L vitamin K, 2 mL/L Tween 80, and 4 mL/L resazurin (0.025%, w:v). Before incubation, vessels were gassed overnight with oxygen-free N_2_ to obtain anaerobic conditions. Carbohydrate substrates were diluted in basal medium (1%, w:v) and filter-sterilized (0.22 μm) and finally added to the corresponding vessels. Nonmodified natural sweeteners were also tested (0.2%, w:v; MV), and fructooligosaccharides (1%, w:v) were used as the positive control. Briefly, 2 mL of fecal inoculum (1:10) was added to each vessel. Negative control cultures consisted of basal medium and inoculum. Fermenters were continually stirred, and the temperature was maintained at 37 °C using a circulating water bath. Culture pH was kept within a range of 6.7 and 6.9 using automated pH controllers to adjust with the addition of NaOH (0.5 M) and HCl (0.5 M) as required (FerMac 260; Electrolab, U.K.). Fermentations were run for a period of 24 h, and samples (2 mL) were obtained from each vessel after 10 and 24 h of fermentation. The samples corresponding to 0 h were only taken from the vessel equivalent to the negative control sample for each donor. Samples collected were centrifuged at 13,000*g* for 10 min to sediment bacteria and other particles and were stored at −20 °C.

### Bacterial Analyses

Bacterial population was quantified by real-time PCR using the estimation of viable bacteria determined by the colony-forming unit (CFU) of each strain (Table 1).^27^

**Table 1:** Group-Specific Primer Set Based on 16S rDNA Sequences

target bacterial group	sequence (5′ to 3′)	product size (bp)	annealing temp (°C)^a^	reference or source
all bacteria	AAACTCAAAKGAATTGACGG	180	60	de Gregoris et al.^49^
	CTCACRRCACGAGCTGAC			
*Lactobacillus*	AGCAGTAGGGAATCTTCCA	341	60	Rinttilä et al.^50^
	CACCGCTACACATGGAG			
*Bacteroides* group *bacteroides – Prevotella – Porphyromonas*	GAAGGTCCCCCACATTG	238	78	Ramirez-Farias et al.^51^
	CGCKACTTGGCTGGTTCAG			
*Bifidobacterium*	CATCCGGCATTACCACCC	523	78	Kok et al.^52^
	CCACCGTTACACCGGGAA			
*Clostidrium coccoides* group	AAATGACGGTACCTGACTAA	438–441	60	Matsuki et al.^53^
	CTTTGAGTTTCATTCTTGCGAA			
*Enterobacteriaceae*	TCAAGGACCAGTGTTCAGTGTC	428	60	Matsuda et al.^54^
	TGCCGTAACTTCGGGAGAAGGCA			
*Enterococcus*	ACCGCGGGTCCATCCATC	115	78	Matsuda et al.^54^
	CCATCAGAAGGGGATAACACTT			
*Atopobium* cluster	GGGTTGAGAGACCGACC	190	60	Matsuki et al.^55^
	CGGRGCTTCTTCTGCAGG			

a The PCR programs were modified from references.

### DNA Extraction

Genomic DNA was extracted from both the pellet (1 mL) of the collected samples from the fermenters and the pure bacterial cultures. Pure bacterial cultures were used to build the corresponding calibration curve (Ct vs CFU/mL). DNA extraction was performed with a NZY Tissue gDNA Isolation kit (NZYTech, Portugal) by adapting the instructions of the manufacturer. The DNA purity and yield were determined by photometry using a NanoDrop (Thermo Scientific NanoDrop OneC). Extracted DNAs were stored at −20 °C until analysis.

### Real-Time PCR Assays

The quantification of the bacterial populations in fecal batch cultures was determined by quantitative real-time PCR of 16S rDNA of targeted bacteria. Table 1 summarizes the oligonucleotide sequences used in the present study and their microbial targets. For each amplification system, the annealing temperature was empirically determined in the laboratory using the respective DNA template isolated from pure cultures from the following species: *Escherichia coli* DSM-6897 (for all bacteria and *Enterobacteria*ceae), *Lactobacillus plantarum* CECT-220 (for *Lactobacillus*), *Enterococcus faecium* CECT-410 (for *Enterococcus*), *Bacteroides xylanisolvens* DSM-23964 (for the Bacteroides group *Bacteroides – Prevotella – Porphyromonas*), *Blautia coccoides* (for the *Clostridium coccoides* group), *Bifidobacterium bifidum* DSM-20456 (for *Bifidobacterium*), and *Atopobium minutum* DSM-20585 (for *the Atopobium* cluster). The primers were commercially synthesized by Eurofins Genomics (Ebersberg, Germany) and Invitrogen Thermo Fisher Scientific (Madrid, Spain).

DNA amplification was performed in 20 μL using a NZY qPCR Green Master Mix (2x) (NZYTech, Portugal) and 0.7 μM of each primer. The reaction was carried out in 384-well optical plates with adhesive sealing and with a ViiA 7 Real-Time PCR System (Applied Biosystems). The thermal cycling program consisted of an initial cycle at 95 °C for 3 min; 40 cycles at 95 °C for 5 s and 60 or 78 °C (Table 1) for 30 s; and finally, two cycles at 95 °C for 15 s.

For the quantification of the target genus or group, the respective standard curve was generated by plotting the Ct values and the corresponding bacterial count (CFU/mL). The bacterial count was determined by extracting the DNA of pure cultures (10^8^ CFU/mL), followed by five 10-fold dilutions and were logarithmized to fit a normal distribution.

### Organic Acid Analysis

Supernatants collected from the centrifuged batch-culture fermented samples were prepared to be analyzed by GC-FID based on the method described by Richardson et al.^28^ A volume of 50 μL of 2-ethylbutyric acid (0.1 M) was added as an internal standard (IS) to 1 mL of a sample. Organic acids were extracted by adding 500 μL of concentrated HCl and 2 mL of diethyl and mixing for 1 min. Samples were centrifuged for 10 min at 2000*g*, and 400 μL of the resulting upper layer (ether layer) was transferred to a GC-capped vial and 50 μL of *N*-(tert-butyldimethysilyl)-*N*-methyltrifluoroacetamine (MTBSTFA) was added. The reaction mixture was left for 72 h at room temperature to ensure full derivatization.

Sample injection was carried out in split mode (100:1) using helium as a carrier gas at a 1.7 mL/min flow rate. The gas chromatograph (Agilent/HP 5890) was equipped with an HP-5MS column (30 m × 0.25 mm) with a 0.25 μm coating (crosslinked (5%-phenyl)-methylpolysiloxane, Hewlett Packard, U.K.). The oven temperature was set with a thermal ramp from 63 °C to 190 °C at a heating rate of 15 °C/min and kept constant for 3 min, and the injector and detector were set at 275 °C. Quantification of lactic acid and other SCFA in the chromatograms was performed using the Agilent ChemStation software (Wilmington, DE) based on the retention times of the respective commercial standards (lactic acid, acetic acid, propionic acid, and butyric acid) ranging between 0.1 and 10 mM.

### Sensory Sweetness Assessment

The sensory analysis was carried out at the Sensory Science Center (Department of Food and Nutritional Sciences, University of Reading, U.K.). The analysis was performed in an air-conditioned (23–24 °C, room temperature) sensory laboratory with individual booths and artificial daylight. The assessment of the sweetness intensity was carried out by a screened and trained sensory panel, which consisted of 10 panelists with between 6 months and 9 years of experience.

The sample of mMV-GOS was assessed for sweetness on a structured line scale against four sucrose standards (0.5, 1.0, 2.0, and 2.6% (w:v)). The average panel ratings for these standards were 10, 35, 75, and 100, respectively, and hence, these four positions were used as anchors to provide a structured scale on which to rate the test sample. The samples were tested in duplicate in two separate tasting sessions. Panelists were given only 0.5 mL of each sample to taste due to sample shortage. Therefore, training additionally focused on ensuring panelists double-blind sip this sample volume from a 30 mL transparent polystyrene cup and allow it to flow over the top of their tongue before swallowing.

An mMV-GOS sample was prepared as 1% (w:v) (weighed to an accuracy of ±0.005 g) in mineral water, stirring over a magnetic plate to ensure thorough sample dispersion. The sample was well dispersed and easily solubilized in water. It was labeled with a random 3-digit code. The sample was tasted and rated as a single (monadic) sample against the sucrose standards; however, other samples were tasted in the same sensory sessions (data not shown). The tasting was approved by the University of Reading Research Ethics Committee (UREC study number 16_19).

### Statistical Analysis

Statistical analysis in bacterial populations and organic acid concentrations was calculated by applying a one-way analysis of variance (ANOVA) for substrate comparisons at the same time of fermentation (10 and 24 h) with Tukey’s test for multiple comparisons, followed by comparison of each substrate at either 10 or 24 h to control 0 h by Student’s *t*-test. The normality of the qPCR data was confirmed by box plots of the logged counts. Differences among samples were judged to be significant at a probability value of *p* ≤ 0.05 (IBM SPSS, Inc. Illinois). The purpose was to quantitatively research whether the new prebiotic-based sweetener behaved just as the positive control and the GOS control samples and differently with the single HIS based on mogroside V (MV control) and the negative control during fermentation, to endorse the potential prebiotic effect. Values were expressed as means ± standard deviation.

The sweetness tasting of the newly synthesized compound mMV-GOS was reported as mean and standard error on the 0–100 structured line scale. A regression line was fit through the mean sweetness against the concentration for the four sucrose standards, where [sweetness = 42.9 (sucrose %w/v) – 9.5] (*R*^2^ = 0.998). This was used to convert the mean sweetness of the mMV-GOS sample into an equivalent sucrose (ES) concentration, and the relative potency was calculated as the ES/mMV-GOS concentration.

## Results

### mMV-GOS Sample Characterization by MALDI-TOF MS

**Figure 1:**
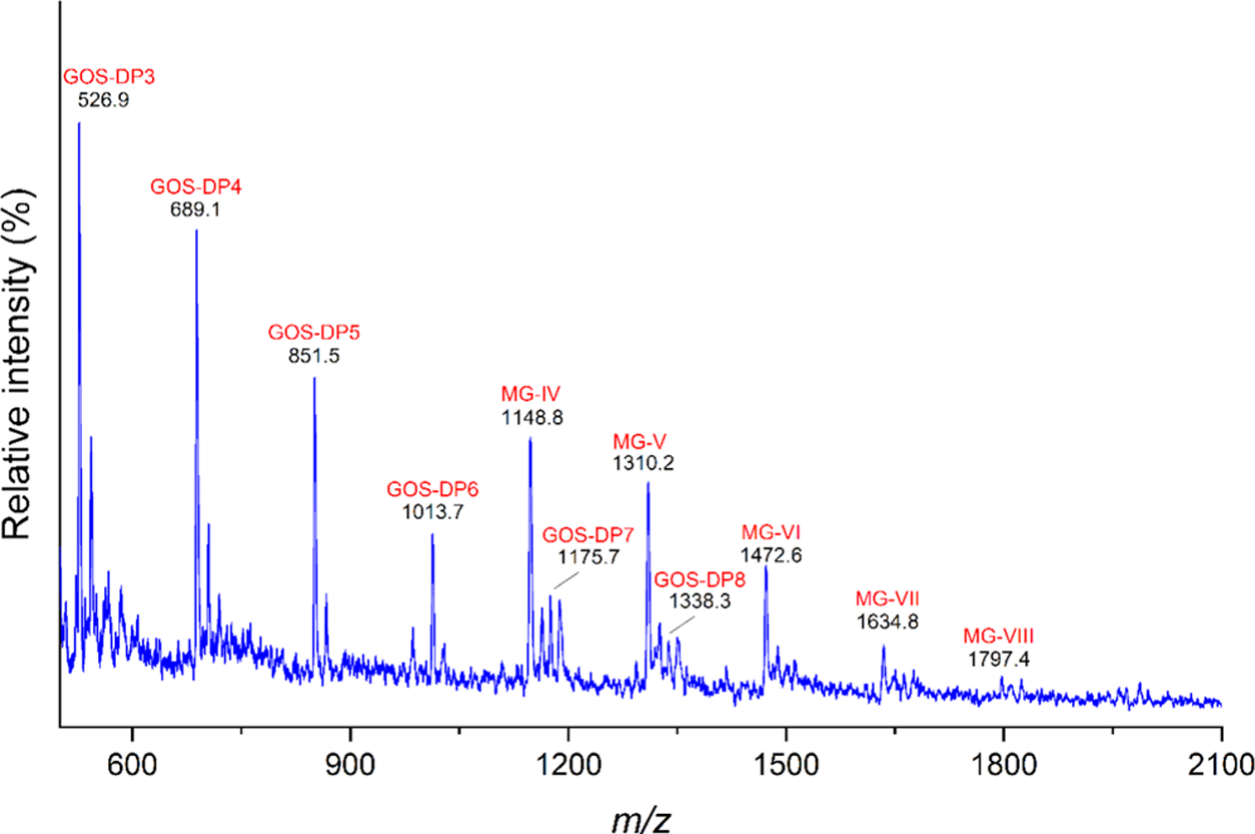
MALDI-TOF profiles of mMV-GOS. Labeled peaks are MG-IV, mogroside IV; MG-V, mogroside V; MG-VI, mogroside V + 1 hexose; MG-VII, mogroside V + 2 hexoses; and MG-VIII, mogroside V + 3 hexoses. The different synthesized oligosaccharides are designated GOS-DP*n*, where n indicates the degree of polymerization (DP).

Figure 1 shows the MALDI-TOF MS spectra of the mMV-GOS sample. The oligomers identified showed a mass difference of 162 u, which corresponds to hexose residues from GOS. The GOS chain length distribution revealed a DP up to 8. The MALDI-TOF MS spectra also show the presence of deglycosylated mogroside V corresponding to *m*/*z* 1148.8, which can be a product of the β-galactosidase reaction^29^ and galactosylated mogroside V with one (*m*/*z* 1472.6), two (*m*/*z* 1634.8), and three (*m*/*z* 1797.4) galactose units.

### Effect of the mMV-GOS Sample on Human Fecal Bacterial Concentrations

**Figure 2:**
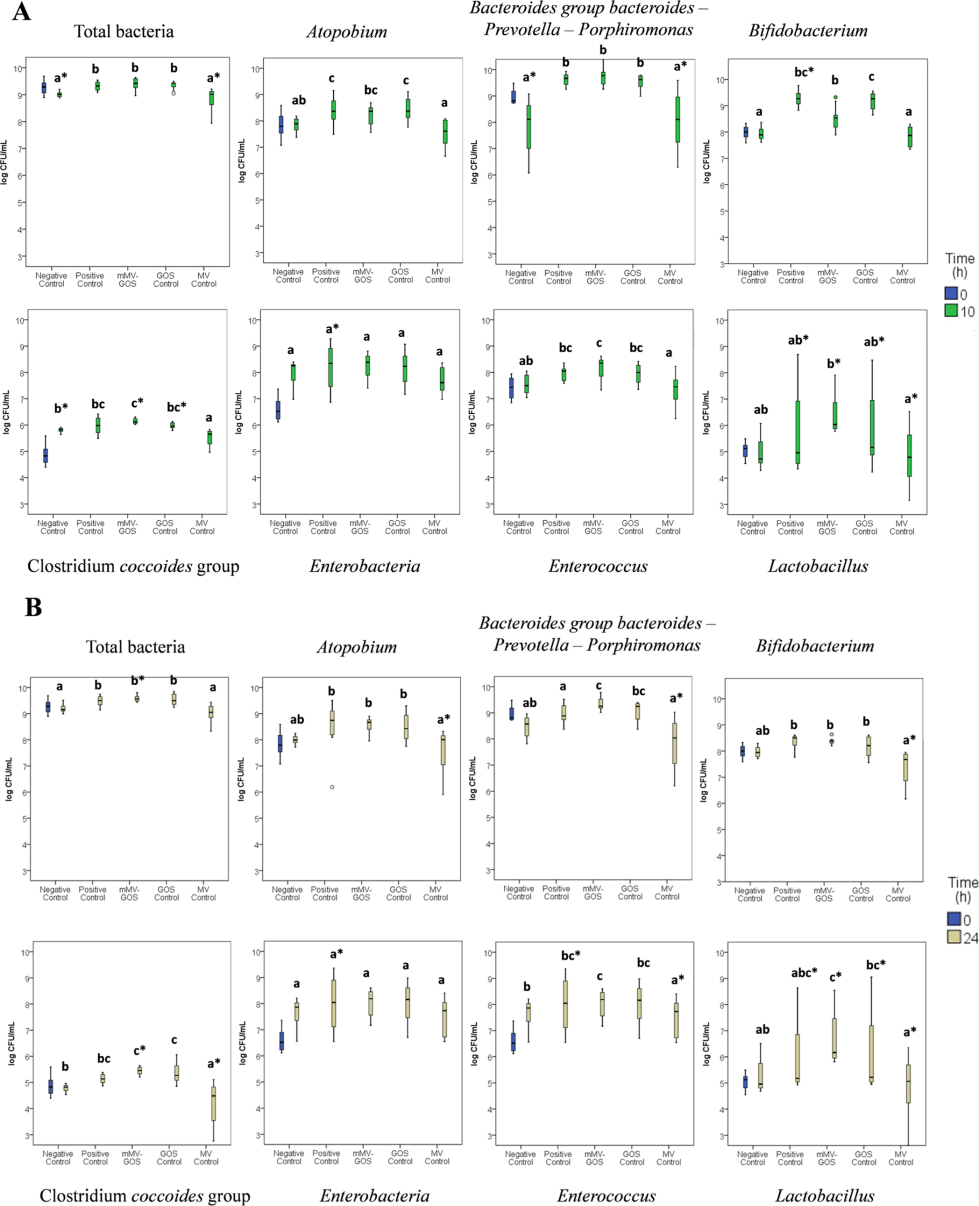
Box plots obtained from the results of the quantitative real-time PCR Ct values for mMV-GOS, obtained from the fecal slurry cultures from four donors after 0 (corresponds to the sample taken from the negative control vessel at the beginning of incubation), (A) 10 h and (B) 24 h for each bacteriological group. Different letters indicate a statistically significant difference between samples at *p* ≤ 0.05 by Tukey’s test and the asterisk symbol indicates statistical differences with respect to the 0-h sample for each bacterial group.

Figure 2A,B show bacterial populations at 0 h with both 10 and 24 h of fermentation for mMV-GOS, GOS control (synthesized under the same reaction conditions as mMV-GOS in the absence of mogrosides), positive control (scFOS), and unmodified mogroside V mixture (MV control). Quantitative data, deviations, and statistical significance are presented in Tables S1 and S2 of the Supplementary material. In general, total bacterial concentrations at 10 and 24 h of fermentation were significantly higher (p ≤ 0.05) for the mMV-GOS, GOS control, and positive control compared to either MV control or negative control (*p* ≤ 0.05) (Tables S1 and S2). The *Atopobium* group significantly increased in the GOS fermentation and positive control sample (10 h), while this significant increase was observed for the mMV-GOS sample after 24 h of fermentation.

For the *Bacteroides - Prevotella - Porphyromonas* group, mMV-GOS did not present significant changes compared to 0 h, except for the bacterial concentrations obtained at 10 and 24 h of fermentation (*p* ≤ 0.05). A similar finding was observed when comparing mMV-GOS to MV sample. Taking into account the values at 10 h of fermentation, the samples GOS and positive control had similar values (9.53 ± 0.29 log CFU/mL and 9.62 ± 0.23 log CFU/mL) to mMV-GOS (9.73 ± 0.31 log CFU/mL), and therefore, the same significances (*p* ≤ 0.05) were found with respect to the negative control and MV control samples (7.84 ± 1.12 log CFU/mL and 8.05 ± 1.18 log CFU/mL).

Significant *Bifidobacterium* population counts were found for mMV-GOS at 10 h compared to the negative control at 10 h as well as for the GOS and positive control samples (*p* ≤ 0.05). This same behavior was noted for the *C. coccoides* group, where mMV-GOS was significantly higher (*p* ≤ 0.05) than the negative control at the initial values (0 h) and at the same time of fermentation (10 and 24 h).

*The Enterobacteriaceae* group increased at 10 and 24 h in the mMV-GOS, GOS, and positive control samples (10 and 24 h; *p* ≤ 0.05) with respect to the negative control; however, no significant differences were found (*p* ≥ 0.05). Among all of the substrates, only the newly synthesized sample (mMV-GOS) significantly increased for *the Enterococcus* group with respect to the negative control sample at the same time of fermentation (*p* ≤ 0.05).

*Lactobacillus* significantly increased in mMV-GOS, GOS, and positive control fermentations after 10 and 24 h (*p* ≤ 0.05) and compared to the beginning of fermentation (0 h) (*p* ≤ 0.05).

### Organic Acid Concentrations during Fermentation with Enzymatically Mogrosides and GOS

Organic acid concentrations are shown in Table 2A for 10 h of fermentation and Table 2B for 24 h of fermentation. Values at the beginning of the fermentation (0 h) corresponding to the negative control sample were used as reference in each table. In general, for unmodified mogroside V, similar behaviors to the negative control were found for the same fermentation times. Acetate was the most abundant organic acid after 10 and 24 h of fermentation in all of the samples. A significant increase of this SCFA was observed for the mMV-GOS (30.78 ± 3.10 mM), GOS (56.11 ± 15.05 mM), and positive control (43.54 ± 6.84 mM) samples at 24 h, where the maximum values were reached (*p* ≤ 0.05). Maximum lactate concentrations increased at 10 h of fermentation for mMV-GOS, scFOS, and GOS, being significantly different to the negative control at the same fermentation time only for these last two substrates. Lactate acid was consumed after 24 h for all fermented samples.

**Table 2:** Mean Organic Acid Concentrations after *In Vitro* Fermentation at (A) 10 and (B) 24 h

A				
	concentration (mM)			
10 h	lactate	acetate	propionate	butyrate
0 h	0.11 ± 0.08	0.73 ± 0.08	0.15 ± 0.07	0.11 ± 0.03
negative control	0.03 ± 0.06a	8.15 ± 1.97a^a^	1.86 ± 0.24a	1.36 ± 0.42a^a^
scFOS (positive control)	20.90 ± 7.65b^a^	46.74 ± 12.13c^a^	6.51 ± 1.91b^a^	1.85 ± 1.23a^a^
GOS control	15.35 ± 4.90b^a^	43.09 ± 11.60c^a^	4.85 ± 1.49b^a^	5.79 ± 1.89b^a^
MV control	0.00 ± 0.00a^a^	7.35 ± 0.92ab^a^	1.57 ± 0.75a^a^	0.83 ± 0.59a^a^
mMV-GOS	2.52 ± 1.69a^a^	20.32 ± 2.56b^a^	9.49 ± 1.44c^a^	2.43 ± 0.73a^a^

B				
	concentration (mM)			
24 h	lactate	acetate	propionate	butyrate
0 h	0.11 ± 0.08	0.73 ± 0.08	0.15 ± 0.07	0.11 ± 0.03
negative control	0.00 ± 0.00a	12.99 ± 1.51a^a^	2.53 ± 0.35a^a^	2.45 ± 0.49a^a^
scFOS (positive control)	0.00 ± 0.00a	43.54 ± 6.84c^a^	6.78 ± 3.50a^a^	6.31 ± 2.91ab^a^
GOS control	0.00 ± 0.00a	56.11 ± 15.05d^a^	13.21 ± 5.15b^a^	13.10 ± 4.99c^a^
MV control	0.00 ± 0.00a	12.28 ± 2.74a^a^	2.41 ± 0.50a^a^	2.13 ± 0.50a^a^
mMV-GOS	0.00 ± 0.00a	30.78 ± 3.10b^a^	14.19 ± 2.16b^a^	7.27 ± 2.22b^a^

a Statistically significant differences from 0 hours at p ≤ 0.05 by Student’s *t*-test are indicated with asterisks in the same column. ^abc^Different letters indicate a statistically significant difference between samples at *p* ≤ 0.05 by Tukey’s test in the same column.

Propionate concentrations presented similar behavior to acetate; a significant increase of propionate was found after 10 and 24 h of fermentation for mMV-GOS and GOS control, with the mMV-GOS sample being the substrate that reached the maximum concentration at both times of fermentation (9.49 ± 1.44 and 14.19 ± 2.16 mM) (p ≤ 0.05). The increased concentration of propionate was lower than acetate but higher when compared with butyrate production. The butyrate concentration at 24 h of fermentation for mMV-GOS (7.27 ± 2.22 mM) was significantly higher than that for the negative control sample, as well as for the GOS control which reached the highest concentration of butyrate when compared to other samples.

### Sweetness of mMV-GOS

A sensory evaluation was performed to find out the effect of the enzymatic modifications on the sweetness of mMV-GOS. The 1% solution of mMV-GOS had a mean sweetness value of 28.6 ± 4.57 (mean out of 100 ± standard error) on the structured line scale. This was equivalent to 0.91 ± 0.3% w/v sucrose, and therefore, an estimated relative potency of 0.9, suggesting that mMV-GOS has a similar sweetness to sucrose. In contrast, pure mogroside V was measured by the same sensory panel to have a relative potency of 188^21^ and is reported in the literature to be approximately 250 x sweeter than sucrose.^17^

## Discussion

There is an increasing demand for natural sweeteners as they are gaining popularity in international markets, including the nutraceutical industries, as a way to offer healthier alternative formulations by significantly reducing the use of sucrose. For this reason and beyond its recognized traditional Chinese medicinal utilization, plant-derived sweeteners from *S. grosvenorii* have recently gained special attention due to their sweet quality, low-calorie characteristics, and being pharmacologically safe.^30^ In addition, numerous studies have demonstrated potential health-promoting effects of mogrosides from luo han guo, including their antioxidant, hepatoprotective, hypoglycemic, immunologic, and anti-inflammatory activities;^31^ however, these findings must be further confirmed in human trials to establish these health-related properties.

Very little is currently known about the impact of mogrosides on the human gut microbiome. Mixtures of mogrosides (MVs), composed mainly of mogroside V, showed no significant effect on the microbiota populations after *in vitro* testing when compared to the negative control;^32^ however, the mogroside doses used were not reported. Recently, Ban et al.^33^ described the effect of supplemented yogurt with mogrosides (from 5 to 30 mg/mL) on the rat intestinal microbiota. The highest concentration of mogrosides used by these authors did not significantly change the population of bifidobacteria and lactobacilli compared with the yogurt control, and there was no impact tested in other bacterial groups. To the best of our knowledge, no previous reports have been published regarding the effect of mogrosides on human gut microbiota.

The sample based on the modified mogrosides with GOS mixtures contains galactosylated and deglycosylated mogroside V and, mainly, the trisaccharide GOS 6′-galactosyl-lactose. This trisaccharide is highly resistant to gastrointestinal digestion when compared to other common GOS such as 3′- and 4′-galactosyl-lactose.^34,35^ This would allow for an increase in the availability of this GOS in the colon which would consequently have a beneficial effect on the colonic microbiota.^36^

The bifidogenic effect of mMV-GOS was similar to that observed for the positive control (scFOS) and GOS which was synthesized in the absence of mogrosides. Both scFOS and GOS are low-molecular-weight oligosaccharides that are rapidly fermented by *Bifidobacterium* species to the concomitant production of lactate and acetate. The bifidobacterial population, as well as the lactate concentration, decreased at 24 h of fermentation. This is due to the utilization of lactate by other bacterial groups by metabolic cross-feeding.^37^ Acetate can also be generated by *the Bacteroides* group, which significantly increased after 10 h of fermentation in the presence of mMV-GOS, GOS, and scFOS. This increase in bifidobacteria and *Bacteroides* group populations after 10 h of fermentation and their concomitant decrease after 24 h have also been observed in FOS samples by different authors.^38,39^ Furthermore, some species of *Bacteroides* are able to produce propionate via succinate pathways and also from lactate, which could also explain the increases in propionic acid.

Lactate can also be generated by other lactic-acid bacteria groups such as *Enterococcus* or *Lactobacillus*. The increases in these two groups of lactic-acid bacteria could be related to the higher concentration of lactate in mMV-GOS when compared with other substrates. It is important to consider the genetic diversity within species that comprise the intestinal microflora such as for this lactic-acid bacterial group, which could explain the large deviation observed for the *Lactobacillus* group in all of the samples.^40,41^*Lactobacillus* is a well-recognized genus with potential beneficial effects on host health.^42^ Recently, the positive effect of the *Enterococcus* genus due to the production of bacteriocins has been reported.^43^

Other propionate and butyrate producers are included in the *C. coccoides* group.^37,44^ This group is formed of different anaerobic species. These species are included in the genera: *Anaerostipes*, *Blautia*, *Coprococcus*, *Clostridium*, *Dorea*, *Eubacterium*, *Ruminococcus*, and *Roseburia*. In general, this group has an important role to play in regulating immunological and nutritional parameters which benefit the health of the host.^45^ Similar to bifidobacteria, the *C. coccoides* group increased significantly after 10 h of fermentation in the GOS and mMV-GOS samples. This could explain the increase of butyrate and propionate after 10 h of fermentation.

It has been reported that mogrosides are metabolized in the intestinal tract, mainly by the complete deglycosylation of mogrosides by the gut microbiota, resulting in the generation of the corresponding aglycone metabolite, mogrol, excreted in feces.^46^ However, the bacterial-mediated deglycosylation does not seem to have an effect on human fecal microbiota composition as its population is not affected by the presence of mogroside V, revealing that the prebiotic effect can be attributed to the presence of GOS.

Our data have shown that mMV-GOS, obtained by the simultaneous synthesis of GOS and modified mogrosides using bacterial β-galactosidases, can generate products with the potential to positively modulate the human fecal microbiota *in vitro*, generating metabolites such as propionate and butyrate that are involved in appetite regulation^47^ and also in tight junction integrity and anti-inflammatory properties that play an important role in improving type II diabetes inflammation processes. The potential prebiotic activity of mMV-GOS is an added benefit in combination with the sweetness profile.

The sweetness intensity of mogrosides is commonly reported in the literature, known to be ∼250 times sweeter than sucrose.^17^ The mMV-GOS had a sweetness that approximated that of sucrose (potency 0.9 ± 0.3). Therefore, one strategy could be to replace sucrose with an equivalent weight of the new mogroside-based sweetener, with the aim of achieving equivalent sweetness, bulking properties, and prebiotic function due to their dietary fiber content (GOS). Previous studies show that a dose between 2 and 15 g/day in adults can exert a prebiotic effect in humans;^24^ on the other hand, mogroside V has been used in doses between 0.6 and 36.4 mg/kg body weight per day; however, the acceptable daily intake (ADI) has not been still approved by some authorities.^48^ The mMV-GOS mixture contains no more than 0.2% w/w of modified mogrosides, which is considerably lower than the maximum dose reported for mogroside V considering a dose of 15 g per day per person. Given the purity of mMV-GOS (>95% of GOS), this novel prebiotic ingredient would correspond to approximately one-half and one-third of the recommended daily sugar consumption by the AHA limits for women (25 g per day) and men (36 g per day), respectively.^23^

In conclusion, these *in vitro* analyses and sweet taste studies suggest that the simultaneous synthesis of modified mogrosides and GOS could exert a prebiotic functionality, which warrants further studies investigating the effects of this novel ingredient under *in vivo* conditions representing physiologically human-relevant exposure scenarios. In this context, these substrates could be considered as a novel candidate prebiotic sweetener by combining sweetness and prebiotic fiber functionality, providing a feasible and innovative approach to reducing the sucrose content in food products.

## Supporting Information Available

Results of quantitative real-time PCR after in vitro fermentation at 10 and 24 h and statistical test data (PDF)

## Abbreviations

GOS: galactooligosaccharides
mMV-GOS: modified mogroside V
MV: unmodified mogroside extracts mainly formed by mogroside V
scFOS: short-chain fructooligosaccharides

## References

[ref1] FAO Carbohydrates in Human Nutrition. Report of a Joint FAO/WHO Expert Consultation FAO Food Nutr. Pap. 1998, 66, 1 140. 9743703

[ref2] Stephen A. M.; Champ M. M. J.; Cloran S. J.; Fleith M.; Van Lieshout L.; Mejborn H.; Burley V. J. Dietary Fibre in Europe: Current State of Knowledge on Definitions, Sources, Recommendations, Intakes and Relationships to Health. Nutr. Res. Rev. 2017, 30, 149–190. 10.1017/S095442241700004X. 28676135

[ref3] Florowska A.; Krygier K.; Florowski T.; Dłuzewska E. Prebiotics as Functional Food Ingredients Preventing Diet-Related Diseases. Food Funct. 2016, 7, 2147–2155. 10.1039/C5FO01459J. 26961814

[ref4] Kumar C. G.; Sripada S.; Poornachandra Y. Status and Future Prospects of Fructooligosaccharides as Nutraceuticals; Elsevier Inc., 2018.

[ref5] Morton G. J.; Meek T. H.; Schwartz M. W. Neurobiology of Food Intake in Health and Disease. Nat. Rev. Neurosci. 2014, 15, 367–378. 10.1038/nrn3745. 24840801 PMC4076116

[ref6] WHO Guideline: Sugars Intake for Adults and Children, WHO Press, World Health Organization, 2015; Vol. 57, pp 1716–1722. 25905159

[ref7] Mela D. J.; MWoolner E. Perspective: Total, Added, or Free? What Kind of Sugars Should We Be Talking About?. Adv. Nutr. 2018, 9, 63–69. 10.1093/advances/nmx020. 29659689 PMC5916432

[ref8] Rippe J. M.; Angelopoulos T. J. Added Sugars and Risk Factors for Obesity, Diabetes and Heart Disease. Int. J. Obes. 2016, 40, S22–S27. 10.1038/ijo.2016.10. 27001643

[ref9] Slavin J. Fiber and Prebiotics: Mechanisms and Health Benefits. Nutrients 2013, 5, 1417–1435. 10.3390/nu5041417. 23609775 PMC3705355

[ref10] Gibson G. R.; Hutkins R.; Sanders M. E.; Prescott S. L.; Reimer R. A.; Salminen S. J.; Scott K. P.; Stanton C.; Swanson K. S.; Cani P. D.; Verbeke K.; Reid G. CONSENSUS The International Scientific Association for Probiotics and Prebiotics (ISAPP). Nat. Publ. Gr. 2017, 14, 491–502. 10.1038/nrgastro.2017.75 28611480

[ref11] Li W.; Wang K.; Sun Y.; Ye H.; Hu B.; Zeng X. Influences of Structures of Galactooligosaccharides and Fructooligosaccharides on the Fermentation in Vitro by Human Intestinal Microbiota. J. Funct. Foods 2015, 13, 158–168. 10.1016/j.jff.2014.12.044.

[ref12] Wang Y.; Dilidaxi D.; Wu Y.; Sailike J.; Sun X.; Nabi X. hua. Composite Probiotics Alleviate Type 2 Diabetes by Regulating Intestinal Microbiota and Inducing GLP-1 Secretion in Db/Db Mice. Biomed. Pharmacother. 2020, 125, 109914 10.1016/j.biopha.2020.109914. 32035395

[ref13] Dominguez A. L.; Rodrigues L. R.; Lima N. M.; Teixeira J. A. An Overview of the Recent Developments on Fructooligosaccharide Production and Applications. Food Bioprocess Technol. 2014, 7, 324–337. 10.1007/s11947-013-1221-6.

[ref14] Hawrelak J. A. Prebiotics, synbiotics and colonic foods. In Textbook of Natural Medicine; Pizzorno J. E.; Murray M. T., Eds.; Elsevier Ltd.: United States, 2020; pp 797–808, 9780323523424.

[ref15] Ruiz-Aceituno L.; Hernandez-Hernandez O.; Kolida S.; Moreno F. J.; Methven L. Sweetness and Sensory Properties of Commercial and Novel Oligosaccharides of Prebiotic Potential. LWT 2018, 97, 476–482. 10.1016/j.lwt.2018.07.038.

[ref16] Martins G. N.; Ureta M. M.; Tymczyszyn E. E.; Castilho P. C.; Gomez-Zavaglia A. Technological Aspects of the Production of Fructo and Enzymatic Synthesis and Hydrolysis. Front. Nutr. 2019, 6, 78 10.3389/fnut.2019.00078. 31214595 PMC6554340

[ref17] Lindley M. G. Natural High-Potency Sweeteners. In Sweeteners and Sugar Alternatives in Food Technology, 2nd ed.; O’Donnell K.; Kearsley M. W., Eds.; John Wiley & Sons, Ltd.: Chichester West Sussex, UK, 2012; pp 185–212.

[ref18] Pawar R. S.; Krynitsky A. J.; Rader J. I. Sweeteners from Plants—with Emphasis on Stevia Rebaudiana (Bertoni) and Siraitia Grosvenorii (Swingle). Anal. Bioanal. Chem. 2013, 405, 4397–4407. 10.1007/s00216-012-6693-0. 23341001

[ref19] Swiader K.; Wegner K.; Piotrowska A.; Tan F. J.; Sadowska A. Plants as a Source of Natural High-Intensity Sweeteners: A Review. J. Appl. Bot. Food Qual. 2019, 92, 160–171. 10.5073/JABFQ.2019.092.022.

[ref20] Yoshikawa S.; Murata Y.; Sugiura M.; Kiso T.; Shizuma M.; Kitahata S.; Nakano H. Transglycosylation OfYoshikawa, Shinichi, Yuji Murata, Masaki Sugiura, Taro Kiso, Motohiro Shizuma, Sumio Kitahata, and Hirofumi Nakano. 2005. “Transglycosylation of Mogroside V, a Triterpene Glycoside in Siraitia Grosvenori, by Cyclodextrin Glucanotransf. J. Appl. Glycosci. 2005, 52, 247–252. 10.5458/jag.52.247.

[ref21] Muñoz-Labrador A.; Azcarate S.; Lebrón-Aguilar R.; Quintanilla-López J. E.; Galindo-Iranzo P.; Kolida S.; Methven L.; Rastall R. A.; Moreno F. J.; Hernandez-Hernandez O. High-Yield Synthesis of Transglycosylated Mogrosides Improves the Flavor Profile of Monk Fruit Extract Sweeteners. J. Agric. Food Chem. 2021, 69, 1011–1019. 10.1021/acs.jafc.0c07267. 33428404

[ref22] Belloir C.; Neiers F.; Briand L. Sweeteners and Sweetness Enhancers. Belloir, C.; Neiers, F.; Briand, L. Sweeteners and sweetness enhancers. Curr. Opin. Clin. Nutr. Metab. Care 2017, 20, 279–285. 10.1097/MCO.0000000000000377. 28399012

[ref23] Johnson R. K.; Appel L. J.; Brands M.; Howard B. V.; Lefevre M.; Lustig R. H.; Sacks F.; Steffen L. M.; Wylie-Rosett J. Dietary Sugars Intake and Cardiovascular Health. Circulation 2009, 120, 1011–1020. 10.1161/CIRCULATIONAHA.109.192627. 19704096

[ref24] Azcarate-Peril M. A.; Ritter A. J.; Savaiano D.; Monteagudo-mera A.; Anderson C. Impact of Short-Chain Galactooligosaccharides on the Gut Microbiome of Lactose-Intolerant Individuals. Proc. Natl. Acad. Sci. U.S.A. 2016, 5, 367–375. 10.1073/pnas.1606722113. PMC5255593 28049818

[ref25] O’Hara S. P.; Hernandez O.; Kolida S. Prebiotic compositions and methods of production thereof. WO2019193358A1, Provided by Sci Finder, 2019 :1923577, 2019 (accessed September 09, 2021).

[ref26] Delgado-Fernandez P.; de Las Rivas B.; Muñoz R.; Jimeno M. L.; Doyagüez E. G.; Corzo N.; Moreno F. J. Biosynthesis of Nondigestible Galactose-Containing Hetero-oligosaccharides by *Lactobacillus plantarum* WCFS1 MelA α-Galactosidase. J. Agric. Food Chem. 2021, 69, 955–965. 10.1021/acs.jafc.0c06417. 33434031

[ref27] Ferreira-Lazarte A.; Moreno F. J.; Cueva C.; Gil-Sánchez I.; Villamiel M. Behaviour of citrus pectin during its gastrointestinal digestion and fermentation in a dynamic simulator (simgi). Carbohydr. Polym. 2019, 207, 382–390. 10.1016/j.carbpol.2018.11.088. 30600020

[ref28] Richardson A. J.; Calder A. G.; Stewart C. S.; Smith A. Simultaneous determination of volatile and non-volatile acidic fermentation products of anaerobes by capillary gas chromatography. Lett. Appl. Microbiol. 1989, 9, 5–8. 10.1111/j.1472-765X.1989.tb00278.x.

[ref29] Wang X.; Gibson G. R.; Costabile A.; Sailer M.; Theis S.; Rastall R. A. Prebiotic Supplementation of in Vitro Fecal Fermentations Inhibits Proteolysis by Gut Bacteria, and Host Diet Shapes Gut Bacterial Metabolism and Response to Intervention. Appl. Environ. Microbiol. 2019, 85, e02749-18 10.1128/AEM.02749-18. 30824442 PMC6495761

[ref30] Gong X.; Chen N.; Ren K.; Jia J.; Wei K.; Zhang L.; Lv Y.; Wang J.; Li M. The Fruits of Siraitia Grosvenorii: A Review of a Chinese Food-Medicine. Front. Pharmacol. 2019, 10, 1400 10.3389/fphar.2019.01400. 31849659 PMC6903776

[ref31] Li C.; Lin L. M.; Sui F.; Wang Z. M.; Huo H. R.; Dai L.; Jiang T. L. Chemistry and Pharmacology of Siraitia Grosvenorii: A Review. Chin. J. Nat. Med. 2014, 12, 89–102. 10.1016/S1875-5364(14)60015-7. 24636058

[ref32] Zhou G.; Peng Y.; Zhao L.; Wang M.; Li X. Biotransformation of Total Saponins in Siraitia Fructus by Human Intestinal Microbiota of Normal and Type 2 Diabetic Patients: Comprehensive Metabolite Identification and Metabolic Profile Elucidation Using LC-Q-TOF/MS. J. Agric. Food Chem. 2017, 65, 1518–1524. 10.1021/acs.jafc.6b04498. 28133964

[ref33] Ban Q.; Liu Z.; Yu C.; Sun X.; Jiang Y.; Cheng J.; Guo M. Physiochemical, Rheological, Microstructural, and Antioxidant Properties of Yogurt Using Monk Fruit Extract as a Sweetener. J. Dairy Sci. 2020, 103, 10006–10014. 10.3168/jds.2020-18703. 32861489

[ref34] Ferreira-Lazarte A.; Gallego-Lobillo P.; Moreno F. J.; Villamiel M.; Hernandez-Hernandez O. In Vitro Digestibility of Galactooligosaccharides: Effect of the Structural Features on Their Intestinal Degradation. J. Agric. Food Chem. 2019, 67, 4662–4670. 10.1021/acs.jafc.9b00417. 30986057

[ref35] Hernández-Hernández O.; Marín-Manzano M. C.; Rubio L. A.; Moreno F. J.; Sanz M. L.; Clemente A. Monomer and Linkage Type of Galacto-Oligosaccharides Affect Their Resistance to Ileal Digestion and Prebiotic Properties in Rats. J. Nutr. 2012, 142, 1232–1239. 10.3945/jn.111.155762. 22649257

[ref36] Hernandez-Hernandez O. In Vitro Gastrointestinal Models for Prebiotic Carbohydrates: A Critical Review. Curr. Pharm. Des. 2019, 25, 3478–3483. 10.2174/1381612825666191011094724. 31604411

[ref37] Scott K. P.; Duncan S. H.; Flint H. J. Dietary Fibre and the Gut Microbiota. Nutr. Bull. 2008, 33, 201–211. 10.1111/j.1467-3010.2008.00706.x.

[ref38] Ho A. L.; Kosik O.; Lovegrove A.; Charalampopoulos D.; Rastall R. A. In Vitro Fermentability of Xylo-Oligosaccharide and Xylo-Polysaccharide Fractions with Different Molecular Weights by Human Faecal Bacteria. Carbohydr. Polym. 2018, 179, 50–58. 10.1016/j.carbpol.2017.08.077. 29111070

[ref39] Likotrafiti E.; Tuohy K. M.; Gibson G. R.; Rastall R. A. An Invitro Study of the Effect of Probiotics, Prebiotics and Synbiotics on the Elderly Faecal Microbiota. Anaerobe 2014, 27, 50–55. 10.1016/j.anaerobe.2014.03.009. 24685554

[ref40] Dubernet S.; Desmasures N.; Guéguen M. A PCR-Based Method for Identification of Lactobacilli at the Genus Level. FEMS Microbiol. Lett. 2002, 214, 271–275. 10.1111/j.1574-6968.2002.tb11358.x. 12351242

[ref41] Heilig H. G. H. J.; Zoetendal E. G.; Vaughan E. E.; Marteau P.; Akkermans A. D. L.; De Vos W. M. Molecular Diversity of Lactobacillus Spp. and Other Lactic Acid Bacteria in the Human Intestine as Determined by Specific Amplification of 16S Ribosomal DNA. Appl. Environ. Microbiol. 2002, 68, 114–123. 10.1128/AEM.68.1.114-123.2002. 11772617 PMC126540

[ref42] Walter J. Ecological Role of Lactobacilli in the Gastrointestinal Tract: Implications for Fundamental and Biomedical Research. Appl. Environ. Microbiol. 2008, 74, 4985–4996. 10.1128/AEM.00753-08. 18539818 PMC2519286

[ref43] Hanchi H.; Mottawea W.; Sebei K.; Hammami R. The Genus Enterococcus: Between Probiotic Potential and Safety Concerns-an Update. Front. Microbiol. 2018, 9, 1791 10.3389/fmicb.2018.01791. 30123208 PMC6085487

[ref44] Hayashi H.; Sakamoto M.; Kitahara M.; Benno Y. Diversity of the *Clostridium coccoides* Group in Human Fecal Microbiota as Determined by 16S RRNA Gene Library. FEMS Microbiol. Lett. 2006, 257, 202–207. 10.1111/j.1574-6968.2006.00171.x. 16553854

[ref45] Kurakawa T.; Ogata K.; Matsuda K.; Tsuji H.; Kubota H.; Takada T.; Kado Y.; Asahara T.; Takahashi T.; Nomoto K. Diversity of Intestinal *Clostridium coccoides* Group in the Japanese Population, as Demonstrated by Reverse Transcription-Quantitative PCR. PLoS One 2015, 10, e0126226 10.1371/journal.pone.0126226. 26000453 PMC4441462

[ref46] Xu F.; Li D. P.; Huang Z. C.; Lu F. L.; Wang L.; Huang Y. L.; Wang R. F.; Liu G. X.; Shang M. Y.; Cai S. Q. Exploring in Vitro, in Vivo Metabolism of Mogroside V and Distribution of Its Metabolites in Rats by HPLC-ESI-IT-TOF-MSn. J. Pharm. Biomed. Anal. 2015, 115, 418–430. 10.1016/j.jpba.2015.07.024. 26280925

[ref47] Louis P.; Flint H. J. Formation of Propionate and Butyrate by the Human Colonic Microbiota. Environ. Microbiol. 2017, 19, 29–41. 10.1111/1462-2920.13589. 27928878

[ref48] Younes M.; Aquilina G.; Engel K.; Fowler P.; Jose M.; Fernandez F.; Rainer G.; Gundert-remy U.; Husøy T.; Mennes W.; Moldeus P.; Peter F.; Oskarsson A.; Shah R.; Waalkens-berendsen I.; Detlef W.; Herman L.; Gott D.; Leblanc J.; Giarola A.; Rincon A. M.; Tard A.; Castle L. Safety of Use of Monk Fruit Extract as a Food Additive in Different Food Categories. EFSA Journal 2019, 17, e05921 10.2903/j.efsa.2019.592. 32626208 PMC7008860

[ref49] Bacchetti De Gregoris T.; Aldred N.; Clare A. S.; Burgess J. G. Improvement of Phylum- and Class-Specific Primers for Real-Time PCR Quantification of Bacterial Taxa. J. Microbiol. Methods 2011, 86, 351–356. 10.1016/j.mimet.2011.06.010. 21704084

[ref50] Rinttilä T.; Kassinen A.; Malinen E.; Krogius L.; Palva A. Development of an Extensive Set of 16S RDNA-Targeted Primers for Quantification of Pathogenic and Indigenous Bacteria in Faecal Samples by Real-Time PCR. J. Appl. Microbiol. 2004, 97, 1166–1177. 10.1111/j.1365-2672.2004.02409.x. 15546407

[ref51] Ramirez-Farias C.; Slezak K.; Fuller Z.; Duncan A.; Holtrop G.; Louis P. Effect of Inulin on the Human Gut Microbiota: Stimulation of *Bifidobacterium* Adolescentis and *Faecalibacterium prausnitzii*. Br. J. Nutr. 2009, 101, 533–540. 10.1017/S0007114508019880. 18590586

[ref52] Kok N.; Roberfroid M.; Robert A.; Delzenne N. Involvement of Lipogenesis in the Lower VLDL Secretion Induced by Oligofructose in Rats. Br. J. Nutr. 1996, 76, 881–890. 10.1079/BJN19960094. 9014656

[ref53] Matsuki T.; Watanabe K.; Fujimoto J.; Miyamoto Y.; Takada T.; Matsumoto K.; Oyaizu H.; Tanaka R. Development of 16S RRNA-Gene-Targeted Group-Specific Primers for the Detection and Identification of Predominant Bacteria in Human Feces. Appl. Environ. Microbiol. 2002, 68, 5445–5451. 10.1128/AEM.68.11.5445-5451.2002. 12406736 PMC129894

[ref54] Matsuda K.; Tsuji H.; Asahara T.; Matsumoto K.; Takada T.; Nomoto K. Establishment of an Analytical System for the Human Fecal Microbiota, Based on Reverse Transcription-Quantitative PCR Targeting of Multicopy RRNA Molecules. Appl. Environ. Microbiol. 2009, 75, 1961–1969. 10.1128/AEM.01843-08. 19201979 PMC2663197

[ref55] Matsuki T.; Watanabe K.; Fujimoto J.; Takada T.; Tanaka R. Use of 16S RRNA Gene-Targeted Group-Specific Primers for Real-Time PCR Analysis of Predominant Bacteria in Mouse Feces. Appl. Environ. Microbiol. 2004, 70, 7220–7228. 10.1128/AEM.70.12.7220-7228.2004. 15574920 PMC535136

