# Supplementary material for: Prebiotic Potential of a New Sweetener Based on Galactooligosaccharides and Modified Mogrosides
Source: J Agric Food Chem. 2022 Jul 13;70(29):9048–56. doi: 10.1021/acs.jafc.2c01363 (PMC9335866; doi:10.1021/acs.jafc.2c01363)
Supplement: Supplementary file 1 — jf2c01363_si_001.pdf [file jf2c01363_si_001.pdf]

SUPPLEMENTARY MATERIAL

Prebiotic potential of a new sweetener based on galactooligosaccharides and modified mogrosides -jf-2022-01363m.R1

Ana Muñoz-Labrador[a], Rosa Lebrón-Aguilar[b], Jesús E. Quintanilla-López[b], Plácido Galindo-Iranzo[b], Silvana M. Azcarate[c], Sofia Kolida[d], Vasiliki Kachrimanidou[e], Virginia Garcia-Cañas[a], Lisa Methven[e], Robert A. Rastall[e], F. Javier Moreno[*a] and Oswaldo Hernandez-Hernandez[a]

[a]Institute of Food Science Research, CIAL (CSIC-UAM), Nicolas Cabrera, 9, 28049 Madrid, Spain

[b]Institute of Physical Chemistry 'Rocasolano' (IQFR-CSIC), Serrano 119, 28006 Madrid, Spain

[c]Institute of Earth and Environmental Sciences of La Pampa (INCITAP), Mendoza 109, L6302EPA Santa Rosa, La Pampa, Argentina

[d]OptiBiotix Health plc, Innovation Centre, Innovation Way, Heslington, York, YO10 5DG, UK

[e]Department of Food and Nutritional Sciences, The University of Reading, PO Box 226, Whiteknights, Reading RG6 6 AP, UK

*Correspondence email and telephone: javier.moreno@csic.es ; +34 910017948

Table of Contents

Table S1. Results of quantitative real-time PCR after *in vitro* fermentation at 0 and 10 h corresponding to **Figure 2A**.

Table S2. Results of quantitative real-time PCR after in vitro fermentation at 0 and 24 h corresponding to **Figure 2B.**

Full set of statistical data.

Table S1. Results of quantitative real-time PCR after *in vitro* fermentation at 0 and 10 h corresponding to **Figure 2A**.

Samples 10 h		All bacteria	Atopobium	Bacteroides	Bifidobacterium	Coccoides	Enterobacteria	Enterococcus	Lactobacillus
0 hours	Mean	9.278	5.060	7.849	8.960	7.987	4.863	6.606	7.404
	N	16	16	12	12	8	12	12	12
	Std. Deviation	0.230	0.297	0.465	0.303	0.264	0.347	0.418	0.393
Negative Control	Mean	9.012**a***	7.851**ab**	7.836**a***	7.937**a**	5.809**b***	7.996**a**	7.519**ab**	4.964**ab**
	N	12	12	12	8	12	12	9	12
	Std. Deviation	0.106	0.273	1.110	0.272	0.078	0.522	0.369	0.650
GOS Control	Mean	9.354**b**	8.458**c**	9.533**b**	9.186**c**	5.982**bc***	8.153**a**	7.952**bc**	6.714**ab***
	N	12	12	12	12	12	12	12	12
	Std. Deviation	0.140	0.442	0.289	0.317	0.111	0.666	0.375	1.105
MV Control	Mean	8.841**a***	7.526**a**	8.053**a***	7.838**a**	5.527**a**	7.707**a**	7.323**a**	4.851**a***
	N	12	12	12	12	12	10	12	12
	Std. Deviation	0.408	0.522	1.184	0.377	0.330	0.511	0.621	1.117
mMV-GOS	Mean	9.389**b**	8.226**bc**	9.733**b**	8.494**b**	6.146**c***	8.236**a**	8.165**c**	6.407**b***
	N	12	12	12	12	12	12	12	12
	Std. Deviation	0.237	0.415	0.315	0.437	0.107	0.487	0.416	0.811
Positive Control	Mean	9.322**b**	8.380**c**	9.625**b**	8.911**bc***	5.965**bc**	8.175**a***	7.948**bc**	5.738**ab***
	N	12	12	12	20	11	12	12	12
	Std. Deviation	0.162	0.505	0.235	0.544	0.335	0.864	0.267	1.746

Different letters indicate statistically significant difference between samples at $p \leq 0.05$ by Tukey's test in the same column
Statistical differences respect to 0 hours sample are indicated by asterisk (*) in the same column

Table S2. Results of quantitative real-time PCR after *in vitro* fermentation at 0 and 24 h corresponding to **Figure 2B**.

Samples 24 h		All bacteria	Atopobium	Bacteroides	Bifidobacterium	Coccoides	Enterobacteria	Enterococcus	Lactobacillus
0 hours	Mean	9.278	5.060	7.849	8.960	7.987	4.863	6.606	7.404
	N	16	16	12	12	8	12	12	12
	Std. Deviation	0.230	0.297	0.465	0.303	0.264	0.347	0.418	0.393
Negative Control	Mean	9.196**a**	8.002**ab**	8.465**ab**	7.965**ab**	4.789**b**	7.669**a**	7.648**b**	5.281**ab**
	N	16	12	12	8	12	12	12	16
	Std. Deviation	0.149	0.157	0.414	0.211	0.142	0.556	0.181	0.724
GOS Control	Mean	9.523**b**	8.483**b**	9.054**bc**	8.164**b**	5.362**c**	8.018**a**	7.936**bc**	6.163**bc∗**
	N	14	12	12	8	12	12	12	15
	Std. Deviation	0.217	0.547	0.410	0.418	0.429	0.808	0.365	1.774
MV Control	Mean	9.028**a**	7.573**a∗**	7.826**a∗**	7.370**a∗**	4.195**a∗**	7.566**a**	6.947**a∗**	4.821**a∗**
	N	16	12	12	8	12	10	12	16
	Std. Deviation	0.285	0.958	1.052	0.737	0.886	0.705	0.885	1.322
mMV-GOS	Mean	9.576**b∗**	8.582**b**	9.333**c**	8.379**b**	5.441**c∗**	8.030**a**	8.325**c**	6.684**c∗**
	N	16	12	12	8	12	12	12	16
	Std. Deviation	0.103	0.279	0.265	0.129	0.155	0.550	0.339	1.105
Positive Control	Mean	9.489**b**	8.557**b**	8.959**bc**	8.365**b**	5.135**bc**	8.006**a∗**	8.011**bc∗**	5.971**abc∗**
	N	16	12	12	8	12	12	12	16
	Std. Deviation	0.186	0.880	0.359	0.301	0.182	1.037	0.157	1.553

Different letters indicate statistically significant difference between samples at $p \leq 0.05$ by Tukey's test in the same column
Statistical differences respect to 0 hours sample are indicated by asterisk (*) in the same column

Statistical Data

1. T TEST

0 hours - Negative Control 10 h

Independent Samples Test

		Levene's Test for Equality of Variances		t-test for Equality of Means						
									95% Confidence Interval of the Difference	
		F	Sig.	t	df	Sig. (2-tailed)	Mean Difference	Std. Error Difference	Lower	Upper
All bacteria (CFU/mL)	Equal variances assumed	5,758	,024	3,703	26	,001	,26599	,07183	,11833	,41364
	Equal variances not assumed			4,079	22,223	,000	,26599	,06522	,13082	,40116
Atopobium (CFU/mL)	Equal variances assumed	3,952	,059	-,017	22	,987	-,00259	,15578	-,32565	,32047
	Equal variances not assumed			-,017	17,774	,987	-,00259	,15578	-,33016	,32498
Bacteroides (CFU/mL)	Equal variances assumed	10,356	,004	3,385	22	,003	1,12400	,33206	,43535	1,81265
	Equal variances not assumed			3,385	12,634	,005	1,12400	,33206	,40451	1,84349
Bifidobacterium (CFU/mL)	Equal variances assumed	,003	,957	,371	14	,716	,04970	,13405	-,23781	,33720
	Equal variances not assumed			,371	13,988	,716	,04970	,13405	-,23784	,33723
Coccoides (CFU/mL)	Equal variances assumed	15,676	,001	-9,205	22	,000	-,94548	,10272	-1,15851	-,73245
	Equal variances not assumed			-9,205	12,104	,000	-,94548	,10272	-1,16907	-,72189
Enterobacteria (CFU/mL)	Equal variances assumed	,775	,388	-7,198	22	,000	-1,39013	,19312	-1,79064	-,98963
	Equal variances not assumed			-7,198	21,008	,000	-1,39013	,19312	-1,79174	-,98853
Enterococcus (CFU/mL)	Equal variances assumed	,160	,693	-,683	19	,503	-,11535	,16882	-,46869	,23800
	Equal variances not assumed			-,690	17,956	,499	-,11535	,16722	-,46672	,23603
Lactobacillus (CFU/mL)	Equal variances assumed	,842	,367	-,034	26	,973	-,00416	,12107	-,25301	,24470
	Equal variances not assumed			-,034	21,839	,973	-,00416	,12361	-,26062	,25231

Independent Samples Test

		Levene's Test for Equality of Variances		t-test for Equality of Means						
									95% Confidence Interval of the Difference	
		F	Sig.	t	df	Sig. (2-tailed)	Mean Difference	Std. Error Difference	Lower	Upper
Acetate (mM)	Equal variances assumed	23,849	,000	-10,629	14	,000	-7,42188	,69827	-8,91952	-5,92424
	Equal variances not assumed			-10,629	7,026	,000	-7,42188	,69827	-9,07180	-5,77196

Lactate (mM)	Equal variances assumed	,468	,505	2,567	14	,022	,08480	,03303	,01396	,15565
	Equal variances not assumed			2,567	12,864	,024	,08480	,03303	,01336	,15624
Butyrate (mM)	Equal variances assumed	295,915	,000	-8,446	14	,000	-1,25111	,14812	-1,56880	-,93341
	Equal variances not assumed			-8,446	7,087	,000	-1,25111	,14812	-1,60050	-,90171
Propionate (mM)	Equal variances assumed	4,567	,051	-19,112	14	,000	-1,70989	,08947	-1,90178	-1,51800
	Equal variances not assumed			-19,112	8,160	,000	-1,70989	,08947	-1,91551	-1,50427

0 hours - mMV-GOS 10 h

Independent Samples Test

		Levene's Test for Equality of Variances		t-test for Equality of Means						
									95% Confidence Interval of the Difference	
		F	Sig.	t	df	Sig. (2-tailed)	Mean Difference	Std. Error Difference	Lower	Upper
All bacteria (CFU/mL)	Equal variances assumed	,003	,954	-1,246	26	,224	-,11098	,08906	-,29404	,07208
	Equal variances not assumed			-1,241	23,484	,227	-,11098	,08942	-,29575	,07379
Atopobium (CFU/mL)	Equal variances assumed	,305	,587	-2,099	22	,048	-,37774	,17997	-,75098	-,00450
	Equal variances not assumed			-2,099	21,715	,048	-,37774	,17997	-,75126	-,00422
Bacteroides (CFU/mL)	Equal variances assumed	,010	,920	-6,135	22	,000	-,77372	,12612	-1,03529	-,51216
	Equal variances not assumed			-6,135	21,971	,000	-,77372	,12612	-1,03531	-,51214
Bifidobacterium (CFU/mL)	Equal variances assumed	1,647	,216	-2,928	18	,009	-,50721	,17322	-,87113	-,14330
	Equal variances not assumed			-3,230	17,906	,005	-,50721	,15703	-,83724	-,17718
Coccoides (CFU/mL)	Equal variances assumed	11,423	,003	-12,229	22	,000	-1,28294	,10491	-1,50051	-1,06536
	Equal variances not assumed			-12,229	13,085	,000	-1,28294	,10491	-1,50943	-1,05644
Enterobacteria (CFU/mL)	Equal variances assumed	,450	,510	-8,788	22	,000	-1,62968	,18545	-2,01428	-1,24508
	Equal variances not assumed			-8,788	21,508	,000	-1,62968	,18545	-2,01479	-1,24457
Enterococcus (CFU/mL)	Equal variances assumed	,000	,991	-4,606	22	,000	-,76104	,16523	-1,10371	-,41837
	Equal variances not assumed			-4,606	21,926	,000	-,76104	,16523	-1,10377	-,41831
Lactobacillus (CFU/mL)	Equal variances assumed	13,591	,001	-6,148	26	,000	-1,34697	,21910	-1,79733	-,89660
	Equal variances not assumed			-5,484	13,229	,000	-1,34697	,24560	-1,87663	-,81731

Independent Samples Test

	Levene's Test for Equality of Variances	t-test for Equality of Means

		F	Sig.	t	df	Sig. (2-tailed)	Mean Difference	Std. Error Difference	95% Confidence Interval of the Difference Lower	Upper
Acetate (mM)	Equal variances assumed	20,300	,001	-21,971	12	,000	-19,59031	,89163	-21,53300	-17,64761
	Equal variances not assumed			-18,768	5,008	,000	-19,59031	1,04380	-22,27215	-16,90846
Lactate (mM)	Equal variances assumed	26,981	,000	-11,576	12	,000	-3,23991	,27988	-3,84972	-2,63010
	Equal variances not assumed			-9,913	5,067	,000	-3,23991	,32683	-4,07672	-2,40309
Butyrate (mM)	Equal variances assumed	23,351	,000	-9,096	12	,000	-2,31962	,25502	-2,87525	-1,76398
	Equal variances not assumed			-7,772	5,015	,001	-2,31962	,29845	-3,08611	-1,55312
Propionate (mM)	Equal variances assumed	24,345	,000	-18,619	12	,000	-9,33431	,50134	-10,42663	-8,24199
	Equal variances not assumed			-15,911	5,018	,000	-9,33431	,58665	-10,84073	-7,82789

0 hours - Positive Control 10 h

Independent Samples Test

		Levene's Test for Equality of Variances		t-test for Equality of Means						
		F	Sig.	t	df	Sig. (2-tailed)	Mean Difference	Std. Error Difference	95% Confidence Interval of the Difference Lower	Upper
All bacteria (CFU/mL)	Equal variances assumed	,980	,331	-,574	26	,571	-,04483	,07808	-,20533	,11567
	Equal variances not assumed			-,603	25,935	,551	-,04483	,07429	-,19754	,10789
Atopobium (CFU/mL)	Equal variances assumed	,023	,881	-2,677	22	,014	-,53101	,19835	-,94235	-,11966
	Equal variances not assumed			-2,677	21,852	,014	-,53101	,19835	-,94252	-,11950
Bacteroides (CFU/mL)	Equal variances assumed	,957	,339	-6,008	22	,000	-,66565	,11079	-,89542	-,43588
	Equal variances not assumed			-6,008	20,717	,000	-,66565	,11079	-,89624	-,43505
Bifidobacterium (CFU/mL)	Equal variances assumed	5,502	,027	-4,559	26	,000	-,92380	,20264	-1,34034	-,50726
	Equal variances not assumed			-6,028	24,697	,000	-,92380	,15326	-1,23964	-,60796
Coccoides (CFU/mL)	Equal variances assumed	,000	,996	-7,729	21	,000	-1,10132	,14249	-1,39764	-,80501
	Equal variances not assumed			-7,742	20,937	,000	-1,10132	,14225	-1,39721	-,80544
Enterobacteria (CFU/mL)	Equal variances assumed	6,284	,020	-5,660	22	,000	-1,56863	,27714	-2,14338	-,99387
	Equal variances not assumed			-5,660	15,892	,000	-1,56863	,27714	-2,15647	-,98079
Enterococcus (CFU/mL)	Equal variances assumed	2,752	,111	-3,963	22	,001	-,54348	,13714	-,82789	-,25907
	Equal variances not assumed			-3,963	19,382	,001	-,54348	,13714	-,83014	-,25683
Lactobacillus (CFU/mL)	Equal variances assumed	26,007	,000	-1,533	26	,137	-,67821	,44226	-1,58729	,23088
	Equal variances not assumed			-1,331	11,479	,209	-,67821	,50958	-1,79409	,43768

Independent Samples Test

		Levene's Test for Equality of Variances		t-test for Equality of Means						
								Std. Error	95% Confidence Interval of the Difference	
		F	Sig.	t	df	Sig. (2-tailed)	Mean Difference	Difference	Lower	Upper
Acetate (mM)	Equal variances assumed	8,710	,011	-10,791	13	,000	-46,01667	4,26437	-55,22928	-36,80406
	Equal variances not assumed			-10,038	6,001	,000	-46,01667	4,58402	-57,23314	-34,80020
Lactate (mM)	Equal variances assumed	16,360	,001	-7,687	14	,000	-20,78798	2,70435	-26,58823	-14,98774
	Equal variances not assumed			-7,687	7,001	,000	-20,78798	2,70435	-27,18250	-14,39347
Butyrate (mM)	Equal variances assumed	16,569	,002	-4,061	12	,002	-1,74340	,42934	-2,67885	-,80794
	Equal variances not assumed			-3,468	5,005	,018	-1,74340	,50268	-3,03516	-,45164
Propionate (mM)	Equal variances assumed	50,642	,000	-9,530	12	,000	-6,35337	,66667	-7,80592	-4,90082
	Equal variances not assumed			-8,141	5,010	,000	-6,35337	,78038	-8,35819	-4,34855

0 hours - GOS Control 10 h

Independent Samples Test

		Levene's Test for Equality of Variances		t-test for Equality of Means						
								Std. Error	95% Confidence Interval of the Difference	
		F	Sig.	t	df	Sig. (2-tailed)	Mean Difference	Difference	Lower	Upper
All bacteria (CFU/mL)	Equal variances assumed	2,871	,102	-1,012	26	,321	-,07625	,07534	-,23110	,07861
	Equal variances not assumed			-1,084	25,095	,289	-,07625	,07035	-,22111	,06862
Atopobium (CFU/mL)	Equal variances assumed	,115	,738	-3,287	22	,003	-,60894	,18524	-,99311	-,22477
	Equal variances not assumed			-3,287	21,941	,003	-,60894	,18524	-,99317	-,22471
Bacteroides (CFU/mL)	Equal variances assumed	,079	,782	-4,739	22	,000	-,57336	,12099	-,82428	-,32244
	Equal variances not assumed			-4,739	21,951	,000	-,57336	,12099	-,82431	-,32241
Bifidobacterium (CFU/mL)	Equal variances assumed	1,078	,313	-8,821	18	,000	-1,19920	,13594	-1,48480	-,91359
	Equal variances not assumed			-9,166	16,965	,000	-1,19920	,13083	-1,47526	-,92313
Coccoides (CFU/mL)	Equal variances assumed	10,975	,003	-10,635	22	,000	-1,11879	,10520	-1,33695	-,90062
	Equal variances not assumed			-10,635	13,212	,000	-1,11879	,10520	-1,34568	-,89189
Enterobacteria (CFU/mL)	Equal variances assumed	1,276	,271	-6,812	22	,000	-1,54664	,22703	-2,01747	-1,07580
	Equal variances not assumed			-6,812	18,517	,000	-1,54664	,22703	-2,02266	-1,07061
Enterococcus (CFU/mL)	Equal variances assumed	,018	,894	-3,494	22	,002	-,54786	,15681	-,87307	-,22266
	Equal variances not assumed			-3,494	21,954	,002	-,54786	,15681	-,87311	-,22262
Lactobacillus (CFU/mL)	Equal variances assumed	19,298	,000	-5,750	26	,000	-1,65439	,28770	-2,24578	-1,06301

	Equal variances not assumed			-5,051	12,199	,000	-1,65439	,32754	-2,36674	-,94204

Independent Samples Test

		Levene's Test for Equality of Variances		t-test for Equality of Means						
								Std. Error	95% Confidence Interval of the Difference	
		F	Sig.	t	df	Sig. (2-tailed)	Mean Difference	Difference	Lower	Upper
Acetate (mM)	Equal variances assumed	20,678	,000	-10,327	14	,000	-42,36062	4,10192	-51,15838	-33,56287
	Equal variances not assumed			-10,327	7,001	,000	-42,36062	4,10192	-52,05992	-32,66132
Lactate (mM)	Equal variances assumed	66,681	,000	-8,800	14	,000	-15,23179	1,73089	-18,94417	-11,51940
	Equal variances not assumed			-8,800	7,003	,000	-15,23179	1,73089	-19,32429	-11,13928
Butyrate (mM)	Equal variances assumed	1282,401	,000	-8,954	10	,000	-5,68046	,63437	-7,09393	-4,26699
	Equal variances not assumed			-6,009	3,001	,009	-5,68046	,94540	-8,68864	-2,67228
Propionate (mM)	Equal variances assumed	25,114	,000	-8,897	14	,000	-4,69919	,52819	-5,83205	-3,56633
	Equal variances not assumed			-8,897	7,031	,000	-4,69919	,52819	-5,94705	-3,45133

0 hours - MV Control 10 h

Independent Samples Test

		Levene's Test for Equality of Variances		t-test for Equality of Means						
								Std. Error	95% Confidence Interval of the Difference	
		F	Sig.	t	df	Sig. (2-tailed)	Mean Difference	Difference	Lower	Upper
All bacteria (CFU/mL)	Equal variances assumed	4,465	,044	3,596	26	,001	,43658	,12143	,18699	,68618
	Equal variances not assumed			3,329	16,212	,004	,43658	,13114	,15888	,71429
Atopobium (CFU/mL)	Equal variances assumed	,117	,736	1,600	22	,124	,32293	,20184	-,09566	,74151
	Equal variances not assumed			1,600	21,718	,124	,32293	,20184	-,09597	,74183
Bacteroides (CFU/mL)	Equal variances assumed	7,177	,014	2,570	22	,017	,90645	,35271	,17496	1,63793
	Equal variances not assumed			2,570	12,438	,024	,90645	,35271	,14094	1,67195
Bifidobacterium (CFU/mL)	Equal variances assumed	4,048	,059	,966	18	,347	,14869	,15400	-,17486	,47224
	Equal variances not assumed			1,037	17,905	,313	,14869	,14333	-,15254	,44992
Coccoides (CFU/mL)	Equal variances assumed	,041	,842	-4,799	22	,000	-,66363	,13829	-,95044	-,37683
	Equal variances not assumed			-4,799	21,944	,000	-,66363	,13829	-,95048	-,37679
Enterobacteria (CFU/mL)	Equal variances assumed	,802	,381	-5,560	20	,000	-1,10144	,19810	-1,51467	-,68822
	Equal variances not assumed			-5,456	17,410	,000	-1,10144	,20188	-1,52661	-,67627
Enterococcus (CFU/mL)	Equal variances assumed	1,379	,253	,382	22	,706	,08094	,21207	-,35885	,52074

		F	Sig.	t	df	Sig. (2-tailed)	Mean Difference	Std. Error Difference	Lower	Upper
	Equal variances not assumed			,382	18,592	,707	,08094	,21207	-,36357	,52546
Lactobacillus (CFU/mL)	Equal variances assumed	9,083	,006	,718	26	,479	,20859	,29057	-,38869	,80587
	Equal variances not assumed			,630	12,174	,540	,20859	,33094	-,51133	,92851

Independent Samples Test

		Levene's Test for Equality of Variances		t-test for Equality of Means						
									95% Confidence Interval of the Difference	
		F	Sig.	t	df	Sig. (2-tailed)	Mean Difference	Std. Error Difference	Lower	Upper
Acetate (mM)	Equal variances assumed	30,869	,000	-20,775	11	,000	-6,62489	,31889	-7,32675	-5,92302
	Equal variances not assumed			-16,045	4,042	,000	-6,62489	,41289	-7,76653	-5,48324
Lactate (mM)	Equal variances assumed	11,993	,004	4,014	13	,001	,11471	,02858	,05298	,17644
	Equal variances not assumed			4,312	7,000	,004	,11471	,02660	,05181	,17761
Butyrate (mM)	Equal variances assumed	9,677	,008	-3,452	13	,004	-,71873	,20823	-1,16859	-,26887
	Equal variances not assumed			-3,212	6,032	,018	-,71873	,22374	-1,26550	-,17196
Propionate (mM)	Equal variances assumed	7,762	,018	-5,472	11	,000	-1,41570	,25870	-1,98510	-,84631
	Equal variances not assumed			-4,227	4,044	,013	-1,41570	,33489	-2,34150	-,48991

0 hours - Negative Control 24 h

Independent Samples Test

		Levene's Test for Equality of Variances		t-test for Equality of Means						
									95% Confidence Interval of the Difference	
		F	Sig.	t	df	Sig. (2-tailed)	Mean Difference	Std. Error Difference	Lower	Upper
All bacteria (CFU/mL)	Equal variances assumed	2,643	,114	1,189	30	,244	,08154	,06860	-,05855	,22164
	Equal variances not assumed			1,189	25,663	,245	,08154	,06860	-,05955	,22264
Atopobium (CFU/mL)	Equal variances assumed	12,766	,002	-1,082	22	,291	-,15352	,14182	-,44764	,14060
	Equal variances not assumed			-1,082	13,483	,298	-,15352	,14182	-,45879	,15175
Bacteroides (CFU/mL)	Equal variances assumed	1,461	,240	3,337	22	,003	,49431	,14812	,18713	,80149
	Equal variances not assumed			3,337	20,169	,003	,49431	,14812	,18550	,80312
Bifidobacterium (CFU/mL)	Equal variances assumed	,077	,786	,181	14	,859	,02160	,11947	-,23463	,27784
	Equal variances not assumed			,181	13,345	,859	,02160	,11947	-,23582	,27902
Coccoides (CFU/mL)	Equal variances assumed	8,223	,009	,688	22	,499	,07447	,10830	-,15014	,29907
	Equal variances not assumed			,688	14,587	,503	,07447	,10830	-,15695	,30588

Enterobacteria (CFU/mL)	Equal variances assumed	,775	,388	-7,198	22	,000	-1,39013	,19312	-1,79064	-,98963
	Equal variances not assumed			-7,198	21,008	,000	-1,39013	,19312	-1,79174	-,98853
Enterococcus (CFU/mL)	Equal variances assumed	10,490	,004	-1,955	22	,063	-,24411	,12483	-,50300	,01478
	Equal variances not assumed			-1,955	15,460	,069	-,24411	,12483	-,50950	,02128
Lactobacillus (CFU/mL)	Equal variances assumed	12,385	,001	-1,131	30	,267	-,22149	,19575	-,62126	,17829
	Equal variances not assumed			-1,131	19,910	,271	-,22149	,19575	-,62994	,18696

Independent Samples Test

		Levene's Test for Equality of Variances		t-test for Equality of Means						
								Std. Error	95% Confidence Interval of the Difference	
		F	Sig.	t	df	Sig. (2-tailed)	Mean Difference	Difference	Lower	Upper
Acetate (mM)	Equal variances assumed	18,335	,001	-22,899	14	,000	-12,26565	,53564	-13,41448	-11,11682
	Equal variances not assumed			-22,899	7,044	,000	-12,26565	,53564	-13,53064	-11,00067
Lactate (mM)	Equal variances assumed	13,838	,002	4,312	14	,001	,11471	,02660	,05765	,17177
	Equal variances not assumed			4,312	7,000	,004	,11471	,02660	,05181	,17761
Butyrate (mM)	Equal variances assumed	13,536	,002	-13,375	14	,000	-2,34359	,17522	-2,71940	-1,96778
	Equal variances not assumed			-13,375	7,062	,000	-2,34359	,17522	-2,75719	-1,92999
Propionate (mM)	Equal variances assumed	8,199	,013	-18,730	14	,000	-2,37503	,12680	-2,64700	-2,10306
	Equal variances not assumed			-18,730	7,557	,000	-2,37503	,12680	-2,67045	-2,07960

0 hours - mMV-GOS 24 h

Independent Samples Test

		Levene's Test for Equality of Variances		t-test for Equality of Means						
									95% Confidence Interval of the Difference	
		F	Sig.	t	df	Sig. (2-tailed)	Mean Difference	Std. Error Difference	Lower	Upper
All bacteria (CFU/mL)	Equal variances assumed	7,600	,010	-4,719	30	,000	-,29788	,06312	-,42679	-,16896
	Equal variances not assumed			-4,719	20,774	,000	-,29788	,06312	-,42924	-,16652
Atopobium (CFU/mL)	Equal variances assumed	4,171	,053	-4,680	22	,000	-,73331	,15668	-1,05824	-,40837
	Equal variances not assumed			-4,680	18,013	,000	-,73331	,15668	-1,06247	-,40415
Bacteroides (CFU/mL)	Equal variances assumed	,334	,569	-3,207	22	,004	-,37306	,11634	-,61433	-,13179
	Equal variances not assumed			-3,207	21,620	,004	-,37306	,11634	-,61458	-,13155
Bifidobacterium (CFU/mL)	Equal variances assumed	2,699	,123	-3,772	14	,002	-,39198	,10392	-,61488	-,16908

		F	Sig.	t	df	Sig. (2-tailed)	Mean Difference	Std. Error Difference	Lower	Upper
	Equal variances not assumed			-3,772	10,165	,004	-,39198	,10392	-,62303	-,16093
Coccoides (CFU/mL)	Equal variances assumed	6,389	,019	-5,258	22	,000	-,57743	,10981	-,80516	-,34969
	Equal variances not assumed			-5,258	15,238	,000	-,57743	,10981	-,81117	-,34369
Enterobacteria (CFU/mL)	Equal variances assumed	,450	,510	-8,788	22	,000	-1,62968	,18545	-2,01428	-1,24508
	Equal variances not assumed			-8,788	21,508	,000	-1,62968	,18545	-2,01479	-1,24457
Enterococcus (CFU/mL)	Equal variances assumed	,671	,421	-6,151	22	,000	-,92081	,14971	-1,23128	-,61034
	Equal variances not assumed			-6,151	21,531	,000	-,92081	,14971	-1,23167	-,60995
Lactobacillus (CFU/mL)	Equal variances assumed	20,455	,000	-5,677	30	,000	-1,62434	,28613	-2,20869	-1,03998
	Equal variances not assumed			-5,677	17,158	,000	-1,62434	,28613	-2,22760	-1,02108

Independent Samples Test

		Levene's Test for Equality of Variances		t-test for Equality of Means						
								Std. Error	95% Confidence Interval of the Difference	
		F	Sig.	t	df	Sig. (2-tailed)	Mean Difference	Difference	Lower	Upper
Acetate (mM)	Equal variances assumed	14,956	,002	-27,383	14	,000	-30,04930	1,09735	-32,40289	-27,69571
	Equal variances not assumed			-27,383	7,010	,000	-30,04930	1,09735	-32,64335	-27,45525
Lactate (mM)	Equal variances assumed	13,838	,002	4,312	14	,001	,11471	,02660	,05765	,17177
	Equal variances not assumed			4,312	7,000	,004	,11471	,02660	,05181	,17761
Butyrate (mM)	Equal variances assumed	19,251	,001	-9,128	14	,000	-7,16525	,78497	-8,84885	-5,48166
	Equal variances not assumed			-9,128	7,003	,000	-7,16525	,78497	-9,02125	-5,30926
Propionate (mM)	Equal variances assumed	13,924	,002	-18,364	14	,000	-14,03637	,76435	-15,67574	-12,39700
	Equal variances not assumed			-18,364	7,015	,000	-14,03637	,76435	-15,84300	-12,22974

0 hours - Positive Control 24 h

Independent Samples Test

		Levene's Test for Equality of Variances		t-test for Equality of Means						
								Std. Error	95% Confidence Interval of the Difference	
		F	Sig.	t	df	Sig. (2-tailed)	Mean Difference	Difference	Lower	Upper
All bacteria (CFU/mL)	Equal variances assumed	,357	,555	-2,849	30	,008	-,21096	,07404	-,36217	-,05974
	Equal variances not assumed			-2,849	28,719	,008	-,21096	,07404	-,36246	-,05946
Atopobium (CFU/mL)	Equal variances assumed	1,154	,294	-2,466	22	,022	-,70861	,28731	-1,30444	-,11277
	Equal variances not assumed			-2,466	16,710	,025	-,70861	,28731	-1,31557	-,10164

Bacteroides (CFU/mL)	Equal variances assumed	,419	,524	,004	22	,997	,00051	,13564	-,28079	,28180
	Equal variances not assumed			,004	21,405	,997	,00051	,13564	-,28124	,28225
Bifidobacterium (CFU/mL)	Equal variances assumed	,182	,676	-2,675	14	,018	-,37834	,14145	-,68171	-,07497
	Equal variances not assumed			-2,675	13,772	,018	-,37834	,14145	-,68218	-,07450
Coccoides (CFU/mL)	Equal variances assumed	4,162	,054	-2,400	22	,025	-,27165	,11319	-,50640	-,03690
	Equal variances not assumed			-2,400	16,632	,028	-,27165	,11319	-,51087	-,03243
Enterobacteria (CFU/mL)	Equal variances assumed	6,284	,020	-5,660	22	,000	-1,56863	,27714	-2,14338	-,99387
	Equal variances not assumed			-5,660	15,892	,000	-1,56863	,27714	-2,15647	-,98079
Enterococcus (CFU/mL)	Equal variances assumed	12,788	,002	-4,970	22	,000	-,60719	,12216	-,86054	-,35384
	Equal variances not assumed			-4,970	14,443	,000	-,60719	,12216	-,86845	-,34593
Lactobacillus (CFU/mL)	Equal variances assumed	28,061	,000	-2,305	30	,028	-,91109	,39526	-1,71833	-,10386
	Equal variances not assumed			-2,305	16,097	,035	-,91109	,39526	-1,74861	-,07358

Independent Samples Test

		Levene's Test for Equality of Variances		t-test for Equality of Means						
									95% Confidence Interval of the Difference	
		F	Sig.	t	df	Sig. (2-tailed)	Mean Difference	Std. Error Difference	Lower	Upper
Acetate (mM)	Equal variances assumed	6,772	,026	-18,653	10	,000	-42,81385	2,29530	-47,92810	-37,69959
	Equal variances not assumed			-12,514	3,000	,001	-42,81385	3,42116	-53,70056	-31,92713
Lactate (mM)	Equal variances assumed	6,590	,028	2,976	10	,014	,11471	,03855	,02882	,20060
	Equal variances not assumed			4,312	7,000	,004	,11471	,02660	,05181	,17761
Butyrate (mM)	Equal variances assumed	13,466	,004	-6,357	10	,000	-6,20452	,97602	-8,37923	-4,02982
	Equal variances not assumed			-4,265	3,000	,024	-6,20452	1,45480	-10,83400	-1,57505
Propionate (mM)	Equal variances assumed	12548,358	,000	-6,763	8	,000	-6,62549	,97968	-8,88464	-4,36633
	Equal variances not assumed			-2,677	1,000	,228	-6,62549	2,47506	-38,05922	24,80824

0 hours - GOS Control 24 h

Independent Samples Test

		Levene's Test for Equality of Variances		t-test for Equality of Means						
									95% Confidence Interval of the Difference	
		F	Sig.	t	df	Sig. (2-tailed)	Mean Difference	Std. Error Difference	Lower	Upper
All bacteria (CFU/mL)	Equal variances assumed	,004	,952	-2,992	28	,006	-,24548	,08205	-,41354	-,07742

	Equal variances not assumed			-3,005	27,836	,006	-,24548	,08170	-,41287	-,07808
Atopobium (CFU/mL)	Equal variances assumed	,154	,699	-3,063	22	,006	-,63474	,20724	-1,06453	-,20495
	Equal variances not assumed			-3,063	21,454	,006	-,63474	,20724	-1,06516	-,20431
Bacteroides (CFU/mL)	Equal variances assumed	1,087	,309	-,641	22	,528	-,09428	,14713	-,39941	,21085
	Equal variances not assumed			-,641	20,273	,529	-,09428	,14713	-,40093	,21236
Bifidobacterium (CFU/mL)	Equal variances assumed	1,561	,232	-1,016	14	,327	-,17754	,17476	-,55238	,19729
	Equal variances not assumed			-1,016	11,822	,330	-,17754	,17476	-,55896	,20387
Coccoides (CFU/mL)	Equal variances assumed	,283	,600	-3,128	22	,005	-,49854	,15939	-,82911	-,16798
	Equal variances not assumed			-3,128	21,078	,005	-,49854	,15939	-,82995	-,16714
Enterobacteria (CFU/mL)	Equal variances assumed	1,276	,271	-6,812	22	,000	-1,54664	,22703	-2,01747	-1,07580
	Equal variances not assumed			-6,812	18,517	,000	-1,54664	,22703	-2,02266	-1,07061
Enterococcus (CFU/mL)	Equal variances assumed	,008	,929	-3,436	22	,002	-,53183	,15477	-,85280	-,21086
	Equal variances not assumed			-3,436	21,881	,002	-,53183	,15477	-,85290	-,21075
Lactobacillus (CFU/mL)	Equal variances assumed	121,438	,000	-2,803	25	,010	-1,41239	,50388	-2,45016	-,37462
	Equal variances not assumed			-2,323	10,304	,042	-1,41239	,60797	-2,76162	-,06315

Independent Samples Test

		Levene's Test for Equality of Variances		t-test for Equality of Means						
									95% Confidence Interval of the Difference	
		F	Sig.	t	df	Sig. (2-tailed)	Mean Difference	Std. Error Difference	Lower	Upper
Acetate (mM)	Equal variances assumed	17,371	,001	-10,555	12	,000	-55,38478	5,24729	-66,81763	-43,95192
	Equal variances not assumed			-9,013	5,000	,000	-55,38478	6,14493	-71,18060	-39,58895
Lactate (mM)	Equal variances assumed	6,590	,028	2,976	10	,014	,11471	,03855	,02882	,20060
	Equal variances not assumed			4,312	7,000	,004	,11471	,02660	,05181	,17761
Butyrate (mM)	Equal variances assumed	19,024	,001	-7,467	12	,000	-12,99439	1,74033	-16,78625	-9,20254
	Equal variances not assumed			-6,376	5,000	,001	-12,99439	2,03804	-18,23323	-7,75556
Propionate (mM)	Equal variances assumed	35,221	,000	-7,380	11	,000	-13,05754	1,76936	-16,95188	-9,16321
	Equal variances not assumed			-5,673	4,001	,005	-13,05754	2,30150	-19,44694	-6,66815

0 hours - MV Control 24 h

Independent Samples Test

	Levene's Test for Equality of Variances		t-test for Equality of Means					
	F	Sig.	t	df	Sig. (2-tailed)	Mean Difference	Std. Error Difference	95% Confidence Interval of the Difference

									Lower	Upper
All bacteria (CFU/mL)	Equal variances assumed	,521	,476	2,724	30	,011	,24952	,09159	,06247	,43656
	Equal variances not assumed			2,724	28,751	,011	,24952	,09159	,06213	,43690
Atopobium (CFU/mL)	Equal variances assumed	8,829	,007	,991	21	,333	,31610	,31904	-,34738	,97957
	Equal variances not assumed			,962	13,910	,352	,31610	,32850	-,38889	1,02108
Bacteroides (CFU/mL)	Equal variances assumed	8,626	,008	3,587	22	,002	1,13393	,31614	,47830	1,78956
	Equal variances not assumed			3,587	12,815	,003	1,13393	,31614	,44995	1,81790
Bifidobacterium (CFU/mL)	Equal variances assumed	6,758	,021	2,227	14	,043	,61669	,27691	,02278	1,21060
	Equal variances not assumed			2,227	8,767	,054	,61669	,27691	-,01227	1,24565
Coccoides (CFU/mL)	Equal variances assumed	7,369	,013	2,434	22	,024	,66847	,27468	,09882	1,23813
	Equal variances not assumed			2,434	14,301	,029	,66847	,27468	,08050	1,25644
Enterobacteria (CFU/mL)	Equal variances assumed	,802	,381	-5,560	20	,000	-1,10144	,19810	-1,51467	-,68822
	Equal variances not assumed			-5,456	17,410	,000	-1,10144	,20188	-1,52661	-,67627
Enterococcus (CFU/mL)	Equal variances assumed	5,104	,034	1,638	22	,116	,45753	,27941	-,12192	1,03699
	Equal variances not assumed			1,638	15,176	,122	,45753	,27941	-,13741	1,05247
Lactobacillus (CFU/mL)	Equal variances assumed	12,201	,002	,705	30	,486	,23886	,33884	-,45315	,93088
	Equal variances not assumed			,705	16,511	,491	,23886	,33884	-,47765	,95538

Independent Samples Test

		Levene's Test for Equality of Variances		t-test for Equality of Means						
								Std. Error	95% Confidence Interval of the Difference	
		F	Sig.	t	df	Sig. (2-tailed)	Mean Difference	Difference	Lower	Upper
Acetate (mM)	Equal variances assumed	43,875	,000	-12,111	12	,000	-11,55655	,95418	-13,63553	-9,47757
	Equal variances not assumed			-10,345	5,007	,000	-11,55655	1,11707	-14,42684	-8,68626
Lactate (mM)	Equal variances assumed	13,838	,002	4,312	14	,001	,11471	,02660	,05765	,17177
	Equal variances not assumed			4,312	7,000	,004	,11471	,02660	,05181	,17761
Butyrate (mM)	Equal variances assumed	46,871	,000	-11,599	12	,000	-2,02005	,17416	-2,39952	-1,64058
	Equal variances not assumed			-9,918	5,033	,000	-2,02005	,20367	-2,54259	-1,49752
Propionate (mM)	Equal variances assumed	27,636	,000	-12,769	12	,000	-2,25600	,17667	-2,64093	-1,87106
	Equal variances not assumed			-10,973	5,148	,000	-2,25600	,20560	-2,77997	-1,73202

2. Oneway ANOVA

Homogeneous Subsets 10 h

Lactobacillus (CFU/mL)

Tukey HSD[a]

Samples	N	Subset for alpha = 0.05 1	2
MV Control	12	4,851014191058348	
Negative Control	12	4,963772886392285	4,963772886392285
Positive Control	12	5,737811188885442	5,737811188885442
GOS Control	12	5,826407199197508	5,826407199197508
mMV-GOS	12		6,406571474126737
Sig.		,336	,053

Means for groups in homogeneous subsets are displayed.

a. Uses Harmonic Mean Sample Size = 12.000.

Atopobium (CFU/mL)

Tukey HSD[a]

Samples	N	Subset for alpha = 0.05 1	2	3
MV Control	12	7,525690569442940		
Negative Control	12	7,851207348497070	7,851207348497070	
mMV-GOS	12		8,226356493725467	8,226356493725467
Positive Control	12			8,379625573157968
GOS Control	12			8,457555759649864
Sig.		,378	,240	,701

Means for groups in homogeneous subsets are displayed.

a. Uses Harmonic Mean Sample Size = 12.000.

All bacteria (CFU/mL)

Tukey HSD[a]

Samples	N	Subset for alpha = 0.05 1	2
MV Control	12	8,841054571499614	
Negative Control	12	9,011652115327436	
Positive Control	12		9,322467417087195
GOS Control	12		9,353885866132599
mMV-GOS	12		9,388617433734767
Sig.		,403	,959

Means for groups in homogeneous subsets are displayed.

a. Uses Harmonic Mean Sample Size = 12.000.

Bacteroides (CFU/mL)

Tukey HSD[a]

Samples	N	Subset for alpha = 0.05 1	2
Negative Control	12	7,835672816040922	
MV Control	12	8,053224494723771	
GOS Control	12		9,533033294724298
Positive Control	12		9,625317897259213
mMV-GOS	12		9,733394389986302
Sig.		,955	,966

Means for groups in homogeneous subsets are displayed.

a. Uses Harmonic Mean Sample Size = 12.000.

Bifidobacterium (CFU/mL)

Tukey HSD[a,b]

Samples	N	Subset for alpha = 0.05		
		1	2	3
MV Control	12	7,838184031949150		
Negative Control	8	7,937179168368507		
mMV-GOS	12		8,494088835089634	
Positive Control	20		8,910675605627613	8,910675605627613
GOS Control	12			9,186069536747803
Sig.		,980	,144	,533

Means for groups in homogeneous subsets are displayed.

a. Uses Harmonic Mean Sample Size = 11.765.

b. The group sizes are unequal. The harmonic mean of the group sizes is used. Type I error levels are not guaranteed.

Coccoides (CFU/mL)

Tukey HSD[a]

Samples	N	Subset for alpha = 0.05		
		1	2	3
MV Control	12	5,526934393724177		
Negative Control	12		5,808782992732025	
Positive Control	12		5,981057420340448	5,981057420340448
GOS Control	12		5,982090132911061	5,982090132911061
mMV-GOS	12			6,146237973021997

Sig.		1,000	,318	,366

Means for groups in homogeneous subsets are displayed.

a. Uses Harmonic Mean Sample Size = 12.000.

Enterobacteria (CFU/mL)

Tukey HSD[a,b]

Samples	N	Subset for alpha = 0.05
		1
MV Control	10	7,707398909060503
Negative Control	12	7,996090944069523
GOS Control	12	8,152592219006237
Positive Control	12	8,174585403091575
mMV-GOS	12	8,235633454784672
Sig.		,274

Means for groups in homogeneous subsets are displayed.

a. Uses Harmonic Mean Sample Size = 11.538.

b. The group sizes are unequal. The harmonic mean of the group sizes is used. Type I error levels are not guaranteed.

Enterococcus (CFU/mL)

Tukey HSD[a,b]

Samples	N	Subset for alpha = 0.05		
		1	2	3
MV Control	12	7,323104089789116		
Negative Control	9	7,519393974773051	7,519393974773051	
Positive Control	12		7,947530326800088	7,947530326800088
GOS Control	12		7,951908080290234	7,951908080290234

SAMPLE	N	1	2	3
mMV-GOS	12			8,165086325578384
Sig.		,813	,134	,750

Means for groups in homogeneous subsets are displayed.

a. Uses Harmonic Mean Sample Size = 11.250.

b. The group sizes are unequal. The harmonic mean of the group sizes is used. Type I error levels are not guaranteed.

Lactate

Tukey HSD[a,b]

		Subset for alpha = 0.05	
SAMPLE	N	1	2
MV Control	7	,0000	
Negative Control	8	,0299	
mMV-GOS	8	2,5160	
GOS Control	8		15,3465
Positive Control	8		20,9027
Sig.		,760	,090

Means for groups in homogeneous subsets are displayed.

a. Uses Harmonic Mean Sample Size = 7.778.

b. The group sizes are unequal. The harmonic mean of the group sizes is used. Type I error levels are not guaranteed.

Acetate

Tukey HSD[a,b]

		Subset for alpha = 0.05		
SAMPLE	N	1	2	3
MV Control	5	7,3509		
Negative Control	8	8,1479	8,1479	
mMV-GOS	6		20,3163	
GOS Control	8			43,0866
Positive Control	7			46,7427
Sig.		1,000	,073	,922

Means for groups in homogeneous subsets are displayed.

a. Uses Harmonic Mean Sample Size = 6.583.

b. The group sizes are unequal. The harmonic mean of the group sizes is used. Type I error levels are not guaranteed.

Propionate

Tukey HSD[a,b]

		Subset for alpha = 0.05		
SAMPLE	N	1	2	3
MV Control	5	1,5679		
Negative Control	8	1,8621		
GOS Control	8		4,8514	
Positive Control	6		6,5056	
mMV-GOS	6			9,4865
Sig.		,994	,179	1,000

Means for groups in homogeneous subsets are displayed.

a. Uses Harmonic Mean Sample Size = 6.383.

b. The group sizes are unequal. The harmonic mean of the group sizes is used. Type I error levels are not guaranteed.

Butyrate

Tukey HSD[a,b]

		Subset for alpha = 0.05	
SAMPLE	N	1	2
MV Control	7	,8263	
Negative Control	8	1,3586	
Positive Control	6	1,8509	
mMV-GOS	6	2,4271	
GOS Control	4		5,7880
Sig.		,061	1,000

Means for groups in homogeneous subsets are displayed.

a. Uses Harmonic Mean Sample Size = 5.874.

b. The group sizes are unequal. The harmonic mean of the group sizes is used. Type I error levels are not guaranteed.

Homogeneous Subsets 24 h

Lactobacillus (CFU/mL)

Tukey HSD[a,b]

Samples	N	Subset for alpha = 0.05		
		1	2	3
MV Control	16	4,820739857823437		
Negative Control	16	5,281090814914272	5,281090814914272	
Positive Control	16	5,970699534762247	5,970699534762247	5,970699534762247
GOS Control	15		6,162822898125116	6,162822898125116
mMV-GOS	16			6,683941798090984
Sig.		,123	,353	,568

Means for groups in homogeneous subsets are displayed.

a. Uses Harmonic Mean Sample Size = 15.789.

b. The group sizes are unequal. The harmonic mean of the group sizes is used. Type I error levels are not guaranteed.

All bacteria (CFU/mL)

Tukey HSD[a,b]

Samples	N	Subset for alpha = 0.05	
		1	2
MV Control	16	9,028123809678400	
Negative Control	16	9,196095441464099	
Positive Control	16		9,488598432780973
GOS Control	14		9,523118180216775
mMV-GOS	16		9,575518226701659
Sig.		,134	,734

Means for groups in homogeneous subsets are displayed.

a. Uses Harmonic Mean Sample Size = 15.556.

b. The group sizes are unequal. The harmonic mean of the group sizes is used. Type I error levels are not guaranteed.

Atopobium (CFU/mL)

Tukey HSD[a]

Samples	N	Subset for alpha = 0.05	
		1	2
MV Control	12	7,573158648059068	
Negative Control	12	8,002136660247876	8,002136660247876
GOS Control	12		8,483355696939896
Positive Control	12		8,557223089821042
mMV-GOS	12		8,581923710833860
Sig.		,489	,197

Means for groups in homogeneous subsets are displayed.

a. Uses Harmonic Mean Sample Size = 12.000.

Bacteroides (CFU/mL)

Tukey HSD[a]

Samples	N	Subset for alpha = 0.05		
		1	2	3
MV Control	12	7,825743353102458		
Negative Control	12	8,465362811663587	8,465362811663587	
Positive Control	12		8,959166511730134	8,959166511730134
GOS Control	12		9,053953091255350	9,053953091255350
mMV-GOS	12			9,332732440379976
Sig.		,062	,102	,507

Means for groups in homogeneous subsets are displayed.

a. Uses Harmonic Mean Sample Size = 12.000.

Bifidobacterium (CFU/mL)

Tukey HSD[a]

Samples	N	Subset for alpha = 0.05	
		1	2
MV Control	8	7,370183764489815	
Negative Control	8	7,965272830155362	7,965272830155362
GOS Control	8		8,164416747349367
Positive Control	8		8,365214292324595
mMV-GOS	8		8,378852942348686
Sig.		,053	,295

Means for groups in homogeneous subsets are displayed.

a. Uses Harmonic Mean Sample Size = 8.000.

Coccoides (CFU/mL)

Tukey HSD[a]

Samples	N	Subset for alpha = 0.05		
		1	2	3
MV Control	12	4,194829136405855		
Negative Control	12		4,788836321934534	
Positive Control	12		5,134953275451775	5,134953275451775
GOS Control	12			5,361845048080892

mMV-GOS	12			5,440729759567866
Sig.		1,000	,355	,481

Means for groups in homogeneous subsets are displayed.

a. Uses Harmonic Mean Sample Size = 12.000.

Enterobacteria (CFU/mL)

Tukey HSD[a,b]

Samples	N	Subset for alpha = 0.05
		1
MV Control	10	7,565842541058049
Negative Control	12	7,668656234938715
Positive Control	12	8,006234287238632
GOS Control	12	8,017991052600117
mMV-GOS	12	8,029665645973198
Sig.		,582

Means for groups in homogeneous subsets are displayed.

a. Uses Harmonic Mean Sample Size = 11.538.

b. The group sizes are unequal. The harmonic mean of the group sizes is used. Type I error levels are not guaranteed.

Enterococcus (CFU/mL)

Tukey HSD[a]

Samples	N	Subset for alpha = 0.05		
		1	2	3
MV Control	12	6,946513466004435		
Negative Control	12		7,648154322410629	
GOS Control	12		7,935876429377603	7,935876429377603

Positive Control	12		8,011238658092438	8,011238658092438
mMV-GOS	12			8,324856478091565
Sig.		1,000	,326	,260

Means for groups in homogeneous subsets are displayed.

a. Uses Harmonic Mean Sample Size = 12.000.

Acetate

Tukey HSD[a,b]

SAMPLE	N	Subset for alpha = 0.05			
		1	2	3	4
MV Control	6	12,2825			
Negative Control	8	12,9916			
mMV-GOS	8		30,7753		
Positive Control	4			43,5398	
GOS Control	6				56,1108
Sig.		1,000	1,000	1,000	1,000

Means for groups in homogeneous subsets are displayed.

a. Uses Harmonic Mean Sample Size = 6.000.

b. The group sizes are unequal. The harmonic mean of the group sizes is used. Type I error levels are not guaranteed.

Propionate

Tukey HSD[a,b]

SAMPLE	N	Subset for alpha = 0.05	
		1	2
MV Control	6	2,4082	
Negative Control	8	2,5273	
Positive Control	2	6,7777	
GOS Control	5		13,2098
mMV-GOS	8		14,1886
Sig.		,104	,977

Means for groups in homogeneous subsets are displayed.

a. Uses Harmonic Mean Sample Size = 4.478.

b. The group sizes are unequal. The harmonic mean of the group sizes is used. Type I error levels are not guaranteed.

Butyrate

Tukey HSD[a,b]

SAMPLE	N	Subset for alpha = 0.05		
		1	2	3
MV Control	6	2,1276		
Negative Control	8	2,4511		
Positive Control	4	6,3121	6,3121	
mMV-GOS	8		7,2728	
GOS Control	6			13,1019
Sig.		,072	,969	1,000

Means for groups in homogeneous subsets are displayed.

a. Uses Harmonic Mean Sample Size = 6.000.

b. The group sizes are unequal. The harmonic mean of the group sizes is used. Type I error levels are not guaranteed.
